# Emergency Department–Based Education and mHealth Empowerment Intervention for Hypertension: The TOUCHED Randomized Clinical Trial

**DOI:** 10.1001/jamacardio.2025.0675

**Published:** 2025-04-23

**Authors:** Heather Prendergast, Spyros Kitsiou, Renee Petzel Gimbar, Sally Freels, Anissa Sanders, Martha Daviglus, Pavitra Kotini-Shah, Barry Carter, Marina Del Rios, Sara Heinert, Shaveta Khosla

**Affiliations:** 1 Department of Emergency Medicine, University of Illinois Chicago; 2 Department of Health Information Sciences, University of Illinois Chicago; 3 College of Pharmacy - Pharmacy Practice, University of Illinois Chicago; 4 School of Public Health, University of Illinois Chicago; 5 Department of Medicine, University of Illinois Chicago; 6 Pharmacy Practice and Science, University of Iowa, Iowa City; 7 Department of Emergency Medicine, University of Iowa, Iowa City; 8 Department of Emergency Medicine, Rutgers–Robert Wood Johnson Medical School, New Brunswick, New Jersey

## Abstract

**Importance:**

Hypertension is a leading risk factor for cardiovascular diseases and is often undiagnosed. Emergency department (ED) visits serve as critical access points within health care and present a unique opportunity for hypertension screening and intervention.

**Objective:**

To evaluate the effectiveness of an Education and mHealth Empowerment (E2) intervention compared with usual care in reducing systolic blood pressure (SBP) among patients with elevated BP discharged from the ED.

**Design, Setting, and Participants:**

This randomized clinical trial enrolled participants who presented to an urban academic medical center ED for any indication and had elevated blood pressure (≥140/90 mm Hg and ≤180/110 mm Hg). Eligible participants who were discharged from the ED were enrolled between February 12, 2019, and March 31, 2023, and were randomized to receive either usual care or the intervention with follow-up visits at 3 and 6 months.

**Interventions:**

Usual care involved standard hypertension discharge instructions with a referral for outpatient follow-up. The E2 intervention involved a 3-prong approach, which included a brief Post-Acute Care Hypertension consultation (PACHT-c) with a clinical pharmacist or an advanced practice nurse, a smartphone-enabled BP monitoring kit (Withings device and mobile app) for daily self-monitoring along with behavior change text messages, and primary care referral.

**Main Outcomes and Measures:**

The primary outcome was the mean change in SBP (mm Hg) from baseline to 6 months.

**Results:**

Of the 574 participants enrolled, mean (SD) age was 51.1 (12.5) years, and 323 (56%) were female; 413 were Black (72%), 115 were Hispanic or Latino (20%), 27 were White (5%), and 19 were other race and ethnicity (3%), which included Asian, American Indian, and other racial or ethnic groups. Of the 413 patients with BP data at 6 months, the E2 intervention group (n = 210) showed a greater mean reduction in SBP (mean difference, 4.9 mm Hg; 95% CI, 0.8-9.0 mm Hg; *P* = .02) compared with the usual-care group (n = 203). A similar proportion of patients achieved BP less than or equal to 140/90 mm Hg at 6 months in the intervention arm (42.9% [90 of 210]) and the control arm (36.9% [75 of 203]; *P* = .22).

**Conclusions and Relevance:**

In this single-center randomized clinical trial, a multicomponent intervention directed at patients in the ED who have elevated BP was associated with greater reduction in SBP at 6 months. Identifying patients who present to the ED with hypertension may be a viable strategy to improve BP management.

**Trial Registration:**

ClinicalTrials.gov Identifier: NCT03749499

## Key Points

**Question:**

Does an Education and mHealth Empowerment (E2) intervention delivered by a multidisciplinary team to patients identified in the emergency department (ED) with high systolic blood pressure (SBP) lead to greater reductions in SBP at 6 months?

**Findings:**

In this randomized clinical trial, among 574 patients with elevated BP enrolled from a single-center ED, those randomized to the E2 intervention had greater reduction in SBP at 6 months compared with those who received standard discharge care.

**Meaning:**

Results suggest that an ED-based E2 intervention may be effective in improving hypertension management compared with standard discharge instructions with outpatient referral.

## Teaser

This randomized clinical trial investigated if an Education and mHealth Empowerment intervention delivered to patients in the emergency department with high systolic blood pressure had greater reductions in systolic blood pressure at 6 months compared with standard discharge instructions with outpatient referral.

## Introduction

Hypertension is a highly prevalent condition affecting over 115 million adults in the US.^1^ Uncontrolled hypertension is an independent risk factor for secondary cardiovascular complications. Among adults treated for hypertension, non-Hispanic Black (20.1%) and Hispanic participants (23%) have a lower prevalence of blood pressure (BP) control than non-Hispanic White participants (31.5%).^2,3^ Early identification and management of hypertension reduces morbidity and mortality^4^; thus, primary and secondary prevention of hypertension remains a public health priority.

There were over 139 million emergency department (ED) visits in 2021.^5^ Prevalence of elevated BP among patients in the ED approached 45%.^6^ Uncontrolled hypertension is more frequently seen in EDs as compared with primary care settings.^7,8,9,10^ Hence, the ED is an important site for hypertension screening,^4,11^ and ED-based screening is feasible.^4,9^ Furthermore, EDs serve as the safety net of the health care system and represent a unique opportunity to engage in secondary prevention of cardiovascular complications stemming from uncontrolled hypertension.^12,13^ The prognostic value of ED-measured hypertension in predicting long-term cardiovascular disease (CVD) outcomes has been established.^13^ The American College of Emergency Physicians recommends that patients with elevated BP in the ED be referred for primary care follow-up.^14^ However, it is not known if interventions in the ED before referral to primary care improve BP outcomes. Additionally, the American College of Cardiology and American Heart Association (AHA) hypertension guidelines give their strongest recommendation (class I level A) for team-based care to improve BP control.^15^

Prior work on designing interventions for patients in the ED^9,16,17^ and communities^18,19^ presenting with elevated BP formed the basis for our 2-component intervention. Ensuring the process flow resulted in minimized extra time in the ED, and avoiding additional patient burden was key to implementing our intervention. A brief education reinforcement with a clinical pharmacist or advanced practice nurse (APN) emphasized any medication changes, BP goals, and nonpharmacological strategies before ED discharge.^13,15,20^

Our ED-based randomized clinical trial was designed to assess the effectiveness of an ED-initiated Education and mHealth Empowerment (E2) intervention and primary care referral program. The primary objective was to evaluate the effectiveness of the E2 intervention compared with usual care in reducing systolic BP (SBP) at 6 months from baseline among patients with elevated BP discharged from the ED. The primary end point was the change in SBP (in millimeters of mercury) from baseline to 6 months. The secondary objectives were to assess the effectiveness of intervention in the following: (1) reducing SBP and diastolic BP (DBP) at 3 months from baseline, (2) reducing DBP at 6 months from baseline, (3) BP control, and (4) follow-up rates at 3 months and 6 months.

## Methods

### Study Setting

The study was approved by the institutional review board at the University of Illinois Chicago. Written informed consent was obtained from participants. The study protocol and details about the study design are outlined in our previously published work (Supplement 1).^21^ Briefly, the Targeting of Uncontrolled Hypertension in the Emergency Department (TOUCHED) trial was a prospective, parallel, 2-arm, randomized clinical trial conducted at a single urban academic center ED in the Midwest. Eligibility for the study included the following: age range of 18 to 75 years, verbal fluency in English or Spanish, at least 2 recorded BP readings between greater than or equal to 140/90 mm Hg and less than or equal to 180/110 mm Hg during the ED visit, and scheduled for ED discharge. The BP criteria were selected based on the hypertension management guidelines^22^ applicable at the design of the study. Patients were excluded if pregnant, planning pregnancy within a year, had impaired decision-making, or could not comprehend the study. Those living outside Chicago or planning to relocate within a year were also ineligible. Recruitment was not based on CVD status. Participants self-reported the following races and ethnicities: Black or African American, Hispanic or Latino, White, and other, which included Asian, American Indian, and other racial or ethnic groups. This study followed the Consolidated Standards of Reporting Trials (CONSORT) reporting guidelines.^23^

Patients were screened and BP measurements were taken in the ED by trained research staff^21^ following the AHA guidelines. Participants were instructed to sit upright, with back supported and feet uncrossed for 5 minutes. The same ED Welch Allyn BP monitor was used to measure BP. After consenting and baseline data collection, participants were randomized to the study arms using a stratified block randomization scheme based on sex (male, female) and baseline BP level (stratum BP ≤140/90 mm Hg and ≥160/100 mm Hg, stratum BP ≥160/100 mm Hg). Blocks of 6 and 8 were randomly ordered, and treatment groups were randomly ordered within a block. Outcome assessors, the primary investigator (H.P.), and the statistician (S.F.) were blinded to the study arm assignment until after the primary analysis was complete. Data were housed in REDCap (Research Electronic Data Capture [Vanderbilt University]). Participants received financial compensation for each of the 2 follow-up visits as well as travel accommodations, which included any combination of bus passes, rideshare services, and parking validation.

#### Treatment Groups

The trial consisted of 2 arms: an intervention arm and a control arm. The control arm received standard discharge instructions on hypertension and a primary care referral recommendation. In addition to the discharge instructions and a facilitated referral for primary care follow-up, the intervention arm involved an Education and mHealth Empowerment (E2) intervention consisting of 2 components: (1) a brief Post-Acute Care Hypertension consultation (PACHT-c) with a clinical pharmacist or APN completed before leaving the ED and (2) a patient-centered mHealth intervention that combined the Withings Bluetooth-enabled BP monitor (BP-801) and Health Mate app with 4 behavior-change text messages per week and daily app notification reminders promoting medication adherence and self-monitoring of BP. The Bluetooth-enabled BP monitors were provided and paired with participants’ smartphones before leaving the ED. Participants who did not have a smartphone were provided with a free study smartphone that included a data and text messaging plan.

Based on previous studies,^9,17^ the lead clinical pharmacist designed the PACHT-c intervention to be standardized and conducted within 10 minutes. The purpose of PACHT-c was to facilitate/reinforce the educational component (including BP goal, nutrition, exercise, smoking cessation, and medication discussion) and initiate or adjust antihypertensive medications as appropriate. Once recruitment resumed during the COVID-19 pandemic, touchscreen tablets were used to perform the PACHT-c intervention while maintaining social distancing. Data from the mobile BP monitor and app were remotely collected, and text messages were delivered using iCardia (an mHealth platform).^24^ Follow-up visits for both arms were performed at 3 months and 6 months and consisted of in-person visits and standardized BP assessments.

### Power and Sample Size

Sample size was estimated by comparing the mean SBP at 6 months using a 2-sample, 2-sided, 5% significance *t* test with 85% statistical power. Although initially a sample size of 770 was estimated, it was then recalculated in 2020 during the temporary pause in enrollment due to the COVID-19 shelter-in-place orders. The sample size recalculation was made in consultation with the data and safety monitoring board to take advantage of the repeated measures design of the study, which improves the statistical power.^25^ An adjusted estimate of the SD corrects for overestimation of type I error but leads to a loss in power.^26^ To account for this, we used 87% power and retained the effect size (6.25 mm Hg) as originally expected, resulting in a new sample size of 570 after adjusting for 25% attrition rate.

### Statistical Analysis

A complete case analysis was conducted excluding people with missing outcome data. Demographics and baseline characteristics were summarized overall and stratified by treatment arm. SBP and DBP were analyzed using mixed-effects multiple linear regression models with an unstructured covariance pattern estimating 6 parameters allowing for correlation within participants across time and assuming independence between participants. Two models were fit predicting each of SBP and DBP as a function of treatment arm and time. Time had 3 categories (baseline, 3 months, and 6 months) coded as 2 contrasts or changes from baseline. The primary outcome, change in SBP at 6 months, was evaluated by testing the interaction between treatment arm and the time contrast change from baseline to 6 months against zero. The secondary outcomes were evaluated by testing the interaction between treatment arm and change from baseline to 3 months predicting SBP, and the interactions between treatment arm and change from baseline to 3 months and from baseline to 6 months predicting DBP. Interaction effects were summarized with interaction terms reflecting differences between change scores and corresponding 95% CIs. Model-based estimates of mean outcomes across time were used to create graphs showing SBP and DBP across time by treatment arm. Binary outcomes, indicators for complete follow-up and for controlled BP (SBP 140 or less and DBP 90 or less) at 3 months and at 6 months after baseline, were analyzed using a χ^2^ test for the effect of treatment arm on each outcome and summarized with odds ratios (ORs) and 95% CIs. In addition to the primary analysis, 2 sensitivity analyses were conducted. For the first sensitivity analysis, controlled BP was reanalyzed with a revised definition of SBP less than or equal to 130 mm Hg and DBP less than or equal to 80 mm Hg to be consistent with the 2017 hypertension management guidelines.^15^ As a second sensitivity analysis, multiple imputations were used to confirm our mixed-model (primary) results. We used multiple imputations (standard number of imputations = 25) to impute missing values. Resulting estimates and corresponding SEs for the main analysis tests, interactions between time and treatment arm, were combined. More information on statistical analyses is available in the eMethods in Supplement 2.

All analyses were completed using SAS, version 9.4 (SAS Institute). Repeated measures linear regression models were fit using PROC MIXED with unstructured variance/covariance. A 2-sided *P* value <.05 was considered statistically significant.

## Results

**Figure 1.**
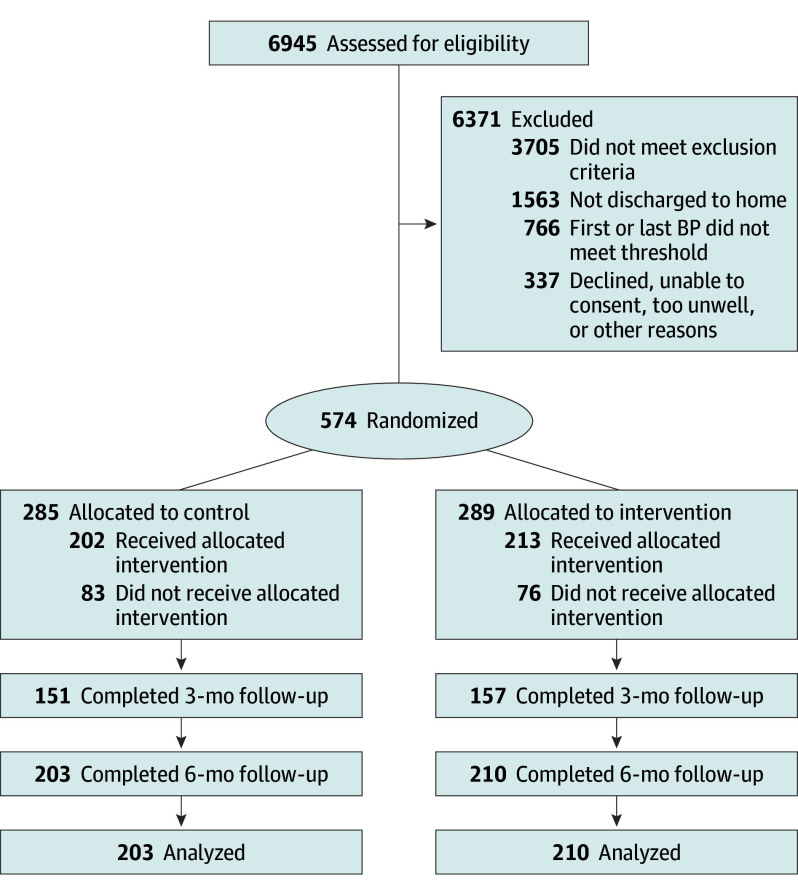
Consolidated Standards of Reporting Trials (CONSORT) Diagram BP indicates blood pressure.

Between February 12, 2019, and March 31, 2023, a total of 574 study participants were enrolled, of which 285 were randomly assigned to the control arm and 289 to the intervention arm (Figure 1). Mean (SD) age for the sample was 51.1 (12.5) years, 323 participants (56%) were female, and 251 participants (44%) were male. Of the total sample, participants self-reported the following races and ethnicities: 413 Black or African American (72%), 115 Hispanic or Latino (20%), 27 White (5%), and 19 were other race and ethnicity (3%). Most of the participants (284 of 574 [50%]) had Medicare/Medicaid insurance. Demographics and baseline characteristics (except use of hypertension medication) were similar between the 2 arms (Table 1).The chief complaint for the ED visit varied and included 139 musculoskeletal (24.2%), 110 cardiovascular (19.2%), 70 neurologic (12.2%), 59 gastrointestinal (10.3%), 41 genitourinary/gynecological (7.2%), 39 otolaryngology related (6.8%), 33 pulmonary (5.8%), 24 dermatologic (4.2%), 9 ocular related (1.6%), 8 endocrine (1.4%), and 44 involved multiple systems or were not system related (8.0%; eg, medication refill, clearances, etc). Percentages are not mutually exclusive.

**Table 1.** Demographics and Baseline Characteristics

Characteristic	Arm	
	Control–usual care (n = 285)	Intervention–E2 intervention (n = 289)
Age, mean (SD), y	51.9 (13.0)	50.3 (11.9)
Sex, No. (%)		
Female	162 (56.8)	161 (55.7)
Male	123 (43.2)	128 (44.3)
Race and ethnicity, No. (%)		
Black or African American	204 (71.6)	209 (72.3)
Hispanic or Latino	56 (19.7)	59 (20.4)
White	17 (6.0)	10 (3.5)
Other^a^	8 (2.7)	11 (3.8)
Type of medical insurance, No. (%)		
Private	69 (24.2)	85 (29.4)
Medicare/Medicaid	149 (52.3)	135 (46.7)
Other	7 (2.4)	8 (2.8)
Missing/refused to answer	60 (21.0)	61 (21.1)
Highest level of education completed, No. (%)		
<HS	47 (16.5)	46 (15.9)
HS graduate or GED	77 (27.0)	70 (24.2)
Some college/associate’s degree	71 (24.9)	76 (26.3)
Bachelor’s degree	19 (6.7)	18 (6.2)
Graduate level degree	11 (3.8)	18 (6.2)
Missing/refused to answer	60 (21.0)	61 (21.1)
How well do you get along on your household income?, No. (%)		
Very well	39 (13.7)	46 (15.9)
Rather well	36 (12.6)	28 (9.7)
Adequately well	108 (37.9)	109 (37.7)
Rather poorly	25 (8.8)	24 (8.3)
Very poorly	17 (6.0)	18 (6.2)
Missing/refused/unknown	60 (21.0)	64 (22.1)
Have a primary care practitioner?, No. (%)		
Yes	225 (78.9)	225 (77.9)
No	55 (19.3)	59 (20.4)
Unknown/missing	5 (1.8)	5 (1.7)
Diabetes, No. (%)		
Yes	80 (28.1)	85 (29.4)
No	198 (69.5)	195 (67.5)
Unknown/missing	7 (2.4)	9 (3.1)
On hypertension treatment?, No. (%)		
Yes	165 (57.9)	143 (49.5)
No	113 (39.6)	137 (47.4)
Unknown/missing	7 (2.5)	9 (3.1)
Ever smoker, No. (%)		
Yes	116 (40.7)	113 (39.1)
No	163 (57.2)	167 (57.8)
Unknown/missing	6 (2.1)	9 (3.1)
Preexisting CHD/CVD or CKD, No. (%)		
Heart failure or myocardial infarction	13 (4.6)	12 (4.2)
Stroke	14 (4.9)	14 (4.8)
ESRD/kidney failure/dialysis	17 (6.0)	17 (5.9)
2 Or more of the above	14 (4.9)	4 (1.4)
No preexisting CVD or CKD	206 (72.3)	215 (74.4)
Unknown/missing	21 (7.4)	27 (9.3)

Abbreviations: CHD, coronary heart disease; CKD, chronic kidney disease; CVD, cardiovascular disease; E2, Education and mHealth Empowerment; ESRD, end-stage renal disease; GED, General Educational Development; HS, high school.

^a^ Other includes participants from all other racial and ethnic groups, excluding Black or African American, Hispanic or Latino, or White.

In the intervention arm, PACHT-c visit was completed for 240 participants, and 125 participants reported taking antihypertensive medication at baseline. Medication was initiated for 91 patients, titrated for 32 patients, and added for 59 patients. The proportions of individuals with complete follow-up were similar between arms at 3 months (54.3% [157 of 289] in intervention arm; 53.0% [151 of 285] in control arm; OR, 1.06; 95% CI, 0.76-1.47; *P* = .75) and at 6 months (72.7% [210 of 289] in intervention arm; 71.2% [203 of 285] in control arm; OR, 1.07; 95% CI, 0.75-1.55; *P* = .70). Three-month follow-up rate was lower overall than the 6-month follow-up rate. At least 4.4% of the sample (25 of 574) missed their 3-month visit around the time that there were restrictions on clinical research due to the COVID-19 pandemic.

### Effect on SBP and DBP

There was a statistically significant decrease in SBP in the intervention arm from baseline to 6 months (interaction [SE], 4.9 [2.1] mm Hg; *t*_572_ = 2.34; *P* = .02) (eTable 1 in Supplement 2). Although both groups experienced a decrease in SBP from baseline to 6 months (Table 2), of the 413 patients with BP data at 6 months, the decrease was larger in E2 intervention arm (−14.3 mm Hg; 210 participants) than in control arm (−9.4 mm Hg; 203 participants) with a mean difference of 4.9 (95% CI, 0.8-9.0) mm Hg.

**Table 2.** Outcomes

Outcome	Arm				Comparison between arms			
	Control–usual care		Intervention–E2 intervention					
	No.	Mean (SD)	No.	Mean (SD)	Interaction (SE)	*df*	*t* Value	*P* value^a^
**Systolic blood pressure**								
At baseline	285	156.5 (13.9)	289	156.4 (13.9)	NA	NA	NA	NA
At 3 mo	151	146.4 (20.1)	157	141.9 (18.8)	NA	NA	NA	NA
At 6 mo	203	146.7 (22.3)	210	141.9 (18.2)	NA	NA	NA	NA
Model-based estimates	**Est (SE)**		**Est (SE)**					
At baseline	156.5 (0.82)		156.5 (0.82)		NA	NA	NA	NA
At 3 mo	146.9 (1.53)		142.7 (1.50)		NA	NA	NA	NA
At 6 mo	147.1 (1.41)		142.2 (1.39)		NA	NA	NA	NA
Change BL to 3 mo	−9.5 (1.60)		−13.7 (1.57)		4.2 (2.2)	572	1.87	.06
Change BL to 6 mo	−9.3 (1.49)		−14.2 (1.47)		4.9 (2.1)	572	2.34	.02^a^
**Diastolic blood pressure**								
At baseline	285	91.7 (11.4)	289	92.6 (12.1)	NA	NA	NA	NA
At 3 mo	151	89.9 (14.2)	157	88.8 (11.9)	NA	NA	NA	NA
At 6 mo	203	90.4 (15.5)	210	88.8 (13.1)	NA	NA	NA	NA
Model-based estimates	**Est (SE)**		**Est (SE)**					
At baseline	91.7 (0.69)		92.6 (0.69)		NA	NA	NA	NA
At 3 mo	90.4 (0.98)		89.6 (0.96)		NA	NA	NA	NA
At 6 mo	90.6 (0.97)		89.2 (0.95)		NA	NA	NA	NA
Change BL to 3 mo	−1.3 (0.94)		−3.0 (0.92)		1.7 (1.3)	572	0.58	.21
Change BL to 6 mo	−1.1 (0.92)		−3.4 (0.90)		2.3 (1.3)	572	1.81	.07
**BP controlled (SBP ≤140 and DBP ≤90)**								
**Outcome**	**No.**	**No. (%)**	**No.**	**No. (%)**	**OR (95% CI)**	**χ^2^**		***P* value**
At 3 mo	151	58 (38.4)	157	80 (51.0)	1.67 (1.06 to 2.62)	4.90		.03^a^
At 6 mo	203	75 (36.9)	210	90 (42.9)	1.28 (0.86 to 1.90)	1.50		.22
**Complete follow-up, No. (%)**								
At 3 mo	285	151 (53.0)	289	157 (54.3)	1.06 (0.76 to 1.47)	0.10		.75
At 6 mo	285	203 (71.2)	289	210 (72.7)	1.07 (0.75 to 1.55)	0.15		.70

Abbreviations: BL, baseline; DBP, diastolic blood pressure; E2, Education and mHealth Empowerment; Est, estimate; NA, not applicable; SBP, systolic blood pressure.

^a^ Indicates significant *P* values.

**Figure 2.**
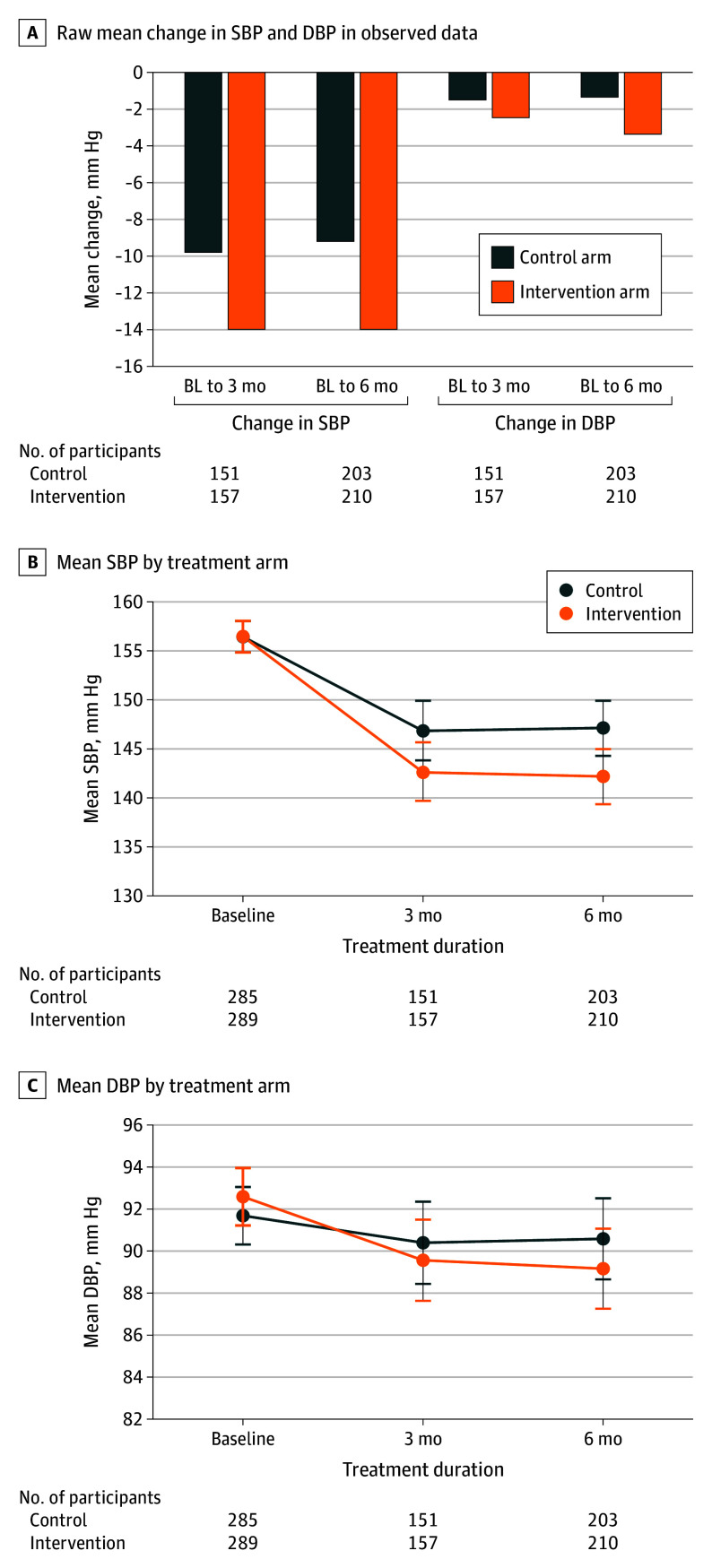
Blood Pressure Change in the 2 Arms A, Observed mean change in systolic blood pressure (SBP) and diastolic blood pressure (DBP) in the observed data. B, Mean SBP by treatment arm (model-based estimates using observed data). C, Mean DBP by treatment arm (model-based estimates using observed data). BL indicates baseline.

The effects on the change in SBP at 3 months and on DBP at 6 months were not significant (interaction [SE], 4.2 [2.2] mm Hg; *t*_572_ = 1.87; *P* = .06 and interaction [SE], 2.3 [1.3] mm Hg; *t*_572_ = 1.81; *P* = .07, respectively). The mean difference in SBP at 3 months (change = −13.8 mm Hg in intervention arm; −9.6 mm Hg in control arm; mean difference = 4.2 mm Hg; 95% CI, −0.2 to 5.6 mm Hg), and DBP at 6 months (change = −3.4 mm Hg in intervention arm; −1.1 mm Hg in control arm; mean difference = 2.3 mm Hg; 95% CI, −0.2 to 4.9 mm Hg) was not statistically significant. There was also no significant difference in DBP at 3 months (change = −3.0 mm Hg in intervention arm; −1.3 mm Hg in control arm; interaction [SE], 1.7 [1.3] mm Hg; *t*_572_ = 0.58; 95% CI, −0.9 to 4.3; *P* = .21). Figure 2 shows the change in BP (SBP and DBP) over time.

After multiple imputation, the mean difference in SBP between the intervention and control arm had consistent findings at 6 months (*t*_442_ = −2.09; *P* = .04) (eTable 3 in Supplement 2).

### Controlled BP

The probability of controlled BP defined as less than or equal to 140/90 mm Hg at 6 months was not significant (42.9% [90 of 210] in intervention arm; 36.9% [75 of 203] in control arm; *P* = .22; OR, 1.28; 95% CI, 0.86-1.90) with similar results when controlled BP was defined as less than or equal to 130/80 mm Hg (eTable 2 in Supplement 2). When examined at 3 months, the probability of controlled BP less than or equal to 140/90 mm Hg was higher in the intervention arm (51.0% [80 of 157]) compared with control arm (38.4% [58 of 151]; OR, 1.67; 95% CI, 1.06-2.62; *P* = .03) (Table 2). Table 3 provides a comparison of those who achieved SBP less than or equal to 140 mm Hg and DBP less than or equal to 90 mm Hg with those who did not achieve it in the intervention arm. Additionally, eTable 4 in Supplement 2 provides the results of BP control across 2 time points (combining results at 3 months and at 6 months) in a single model for each outcome.

**Table 3.** Blood Pressure Controlled vs Not Controlled at 6 Months^a^

Controlled blood pressure defined as SBP ≤140 and DBP ≤90	Blood pressure	
	Controlled (n = 90)	Not controlled (n = 120)
Age		
Mean (SD)	48.5 (13.4)	49.9 (11.5)
Sex, No. (%)		
Male	27 (30.0)	64 (53.3)
Female	63 (70.0)	56 (46.7)
Race and ethnicity, No. (%)		
Black or African American	59 (65.6)	99 (82.5)
Hispanic or Latino	24 (26.7)	18 (15.0)
White	3 (3.3)	3 (2.5)
Other^b^	4 (4.4)	0
Type of medical insurance, No. (%)		
Private	26 (28.8)	34 (28.3)
Medicare/Medicaid	47 (52.2)	60 (50.0)
Other	0 (0.0)	3 (2.5)
Missing/refused to answer	17 (18.9)	23 (19.2)
Highest level of education completed, No. (%)		
<HS	20 (22.2)	16 (13.3)
HS graduate or GED	15 (16.7)	34 (28.3)
Some college/associate’s degree	24 (26.7)	32 (26.7)
Bachelor’s degree	7 (7.8)	6 (5.0)
Graduate level degree	7 (7.8)	9 (7.5)
Missing/refused to answer	17 (18.9)	23 (19.2)
How well do you get along on your household income?, No. (%)		
Very well	13 (14.4)	15 (12.5)
Rather well	9 (10.0)	16 (13.3)
Adequately well	38 (42.2)	42 (35.0)
Rather poorly	8 (8.9)	15 (12.5)
Very poorly	5 (5.6)	8 (6.7)
Missing/refused/unknown	17 (18.9)	24 (20.0)
Have a primary care practitioner?, No. (%)		
Yes	79 (87.8)	93 (77.5)
No	11 (12.2)	27 (22.5)

Abbreviations: DBP, diastolic blood pressure; E2, Education and mHealth Empowerment; GED, General Educational Development; HS, high school; SBP, systolic blood pressure.

^a^ In the intervention arm only, n = 210.

^b^ Other includes participants from all other racial and ethnic groups, excluding Black or African American, Hispanic or Latino, or White.

In the intervention arm, the Bluetooth-enabled BP monitor recorded the SBP, DBP, and heart rate. For 232 participants, the mean (SD) number of recordings for each metric per participant was 70 (93). Among these 232 participants, recordings were done at least 1 day per week for a mean (SD) of 13.8 (12.5) weeks (median [IQR], 9 [3-24] weeks), and 22% of the participants (51 of 232) adhered to monitoring by recording at least 1 day per week (for ≥26 weeks).

## Discussion

In a patient population with elevated BP identified in the ED, a multicomponent BP intervention (E2) was more effective than usual care in reducing SBP from baseline to 6 months. These findings are clinically significant as they suggest that identifying patients in the ED for more targeted interventions could play an important role in the continuum of hypertension care, particularly for patients who may have less access to regular outpatient care and rely on the ED for routine care.

Our findings suggest that initiating or adjusting antihypertensive medications after an ED visit where high BP is observed may optimize BP control in follow-up, providing a bridge to ongoing care. A major component of the intervention was the management provided by clinical pharmacists or APNs embedded in the ED. In our study, approximately 80% of the interventions were provided by clinical pharmacists. Previous systematic reviews have estimated that team-based interventions can reduce SBP between 4 and 10 mm Hg as well as improved BP control.^20,27,28,29^ Our findings in the ED are consistent with findings in other primary care settings.

It is notable that the control arm in our study had a reduction in both SBP and DBP at 3 months, which was sustained to 6 months. Participants in the control arm, now aware of their elevated BP and given discharge instructions, may have had improved BP management due to accountability from anticipated follow-up.

The E2 intervention or ED-based interventions may also help to address CVD-related disparities that disproportionately affect minoritized Black and Latino communities^30,31,32^ by promoting self-monitoring and awareness and focusing on barriers to care. A population-based primary prevention strategy that achieves a 10% reduction in mean BP and cholesterol could reduce the incidence of CVD by up to 45%.^33^ Implementing interventions like E2 on a large scale could significantly advance public health goals. E2 intervention integrates educational and empowerment components, fostering patient engagement and self-efficacy, which are essential for chronic disease management. Traditional care models, which often identify individuals at an advanced stage, may be inadequate for early CVD prevention. E2 intervention represents a new approach, extending public health initiatives beyond conventional settings, which is crucial given the AHA’s recommendation for a multidisciplinary approach to manage asymptomatic hypertension in EDs, particularly for patients lacking reliable outpatient care.^34,35^

This study used consumer-grade mHealth technology, which is accessible and often covered by health savings accounts, making it potentially scalable to other settings. The intervention’s effects were evident at both 3 and 6 months, highlighting its sustained impact and the potential to reduce hypertension-related disparities, especially in underserved populations. The findings advocate for the incorporation of educational and empowering interventions into standard ED practices, similar to smoking cessation protocols.

Future studies will explore the long-term effects of ED-based interventions and identify attributes linked to improved BP management. Insights from this study could refine ED interventions for broader application in multicenter trials. A cost-effectiveness analysis would help quantify the economic benefits of such interventions, and a factorial trial could evaluate the individual and combined effects of intervention components.

Despite COVID-19 pandemic–related challenges, the study maintained a high retention rate at 6 months, using multiple outreach strategies to minimize losses. Notably, trial participants were predominantly Black and Latino patients, who are typically underrepresented in clinical trials.^36^

### Limitations

Our study had several limitations. Recruitment and follow-up coincided with peak of the COVID-19 pandemic, leading to fewer research associates, patient reluctance, and potential selection bias due to convenience sampling. Additionally, missing follow-up data could have diminished the statistical power of our findings with approximately 70% completing follow-up for the primary outcome at 6 months. However, there was no significant imbalance in loss to follow-up between the intervention and control groups. Moreover, the findings between complete case analysis and imputed data analysis were similar. The generalizability of our findings may be restricted given that this was an urban single-center study. In addition, the primary outcome at 6 months represents a short-term follow-up, and differences in longer-term CVD outcomes were not assessed. Finally, the study did not monitor changes in BP treatment in the control group after discharge, thereby limiting our understanding of differences in antihypertensive treatment regimens between arms.

## Conclusions

Results of the TOUCHED randomized clinical trial suggest that a multicomponent Education and Empowerment (E2) intervention initiated in the ED may present a viable and effective strategy for reducing SBP in patients with elevated BP who are discharged from the ED. These findings underscore the critical importance of implementing targeted interventions at key points during health care contact, such as the ED setting with a multidisciplinary team. Such interventions not only address immediate health needs but may also contribute to broader public health objectives with the potential to improve hypertension management. Future research should include multicenter evaluation of this intervention, examine long-term health outcomes, and explore the patient’s perspective.

## Notes

Supplement 1.
Trial Protocol.

Supplement 2.
eMethods.
**eTable 1.** Results for the Interactions Between Time and Treatment Arm Predicting Systolic and Diastolic Blood Pressure Using Observed Data **eTable 2.** Controlled Blood Pressure (Defined as SBP ≤130 and DBP ≤80) by Treatment Arm **eTable 3.** Multiple Imputation Results **eTable 4.** Generalized Linear Models for Binary Outcomes Across 2 Time Points

Supplement 3.
Data Sharing Statement.

## References

[1] Jaeger BC, Chen L, Foti K, . Hypertension statistics for US Adults: an open-source web application for analysis and visualization of National Health and Nutrition Examination Survey Data. Hypertension. 2023;80(6):1311-1320. doi:10.1161/HYPERTENSIONAHA.123.20900 37082970 PMC10424908

[2] Million Hearts. Estimated hypertension prevalence, treatment, and control among US adults. Accessed November 1, 2024. https://millionhearts.hhs.gov/data-reports/hypertension-prevalence.html

[3] Ogunniyi MO, Commodore-Mensah Y, Ferdinand KC. Race, ethnicity, hypertension, and heart disease: JACC focus seminar 1/9. J Am Coll Cardiol. 2021;78(24):2460-2470. doi:10.1016/j.jacc.2021.06.017 34886968

[4] Scott RL, Cummings GE, Newburn-Cook C. The feasibility and effectiveness of emergency department–based hypertension screening: a systematic review. J Am Acad Nurse Pract. 2011;23(9):493-500. doi:10.1111/j.1745-7599.2011.00636.x 21899644

[5] Cairns C, Kang K. National Hospital Ambulatory Medical Care Survey: 2021 Emergency Department Summary Tables. National Center for Health Statistics; 2023.

[6] Miller J, McNaughton C, Joyce K, Binz S, Levy P. Hypertension management in emergency departments. Am J Hypertens. 2020;33(10):927-934. 32307541 10.1093/ajh/hpaa068 PMC7577644

[7] Levy PD, Cline D. Asymptomatic hypertension in the emergency department: a matter of critical public health importance. Acad Emerg Med. 2009;16(11):1251-1257. doi:10.1111/j.1553-2712.2009.00512.x 19845553

[8] Baumann BM, Cline DM, Pimenta E. Treatment of hypertension in the emergency department. J Am Soc Hypertens. 2011;5(5):366-377. doi:10.1016/j.jash.2011.05.002 21719370

[9] Prendergast H, Del Rios M, Durazo-Arvizu R, . Effect of an emergency department education and empowerment intervention on uncontrolled hypertension in a predominately minority population: the AHEAD2 randomized clinical pilot trial. J Am Coll Emerg Physicians Open. 2021;2(2):e12386. doi:10.1002/emp2.12386 33718921 PMC7926004

[10] Chan SS, Graham CA, Rainer TH. Hypertension in the emergency department. Curr Hypertens Rep. 2016;18(5):37. doi:10.1007/s11906-016-0647-4 27072830

[11] Tilman K, DeLashaw M, Lowe S, Springer S, Hundley S, Counselman FL. Recognizing asymptomatic elevated blood pressure in ED patients: how good (bad) are we? Am J Emerg Med. 2007;25(3):313-317. doi:10.1016/j.ajem.2006.09.007 17349906

[12] Ong Eng Hock M, Ornato JP, Cosby C, Franck T. Should the emergency department be society’s health safety net? J Public Health Policy. 2005;26(3):269-281. doi:10.1057/palgrave.jphp.3200028 16167554

[13] Reynard C, van den Berg P, Oliver G, . The prognostic value of emergency department measured hypertension: a systematic review and meta-analysis. Acad Emerg Med. 2022;29(3):344-353. doi:10.1111/acem.14324 34553441

[14] Karras DJ, Kruus LK, Cienki JJ, . Evaluation and treatment of patients with severely elevated blood pressure in academic emergency departments: a multicenter study. Ann Emerg Med. 2006;47(3):230-236. doi:10.1016/j.annemergmed.2005.11.001 16492489

[15] Whelton PK, Carey RM, Aronow WS, . 2017 ACC/AHA/AAPA/ABC/ACPM/AGS/APhA/ASH/ASPC/NMA/PCNA guideline for the prevention, detection, evaluation, and management of high blood pressure in adults: a report of the American College of Cardiology/American Heart Association Task Force on Clinical Practice Guidelines. J Am Coll Cardiol. 2018;71(19):e127-e248. doi:10.1016/j.jacc.2017.11.006 29146535

[16] Prendergast HM, Colla J, Del Rios M, Marcucci J, Schulz R, O’Neal T. Playing a role in secondary prevention in the ED: longitudinal study of patients with asymptomatic elevated blood pressures following a brief education intervention: a pilot study. Public Health. 2015;129(5):604-606. doi:10.1016/j.puhe.2015.02.001 25796291 PMC4721226

[17] Prendergast HM, Del Rios M, Petzel-Gimbar R, . A hypertension emergency department intervention aimed at decreasing disparities: design of a randomized clinical trial. Contemp Clin Trials. 2018;64:1-7. doi:10.1016/j.cct.2017.11.009 29128648 PMC5837806

[18] Prendergast HM, Escobar-Schulz S, Del Rios M, . Community targeting of uncontrolled hypertension: results of a hypertension screening and education intervention in community churches serving predominantly racial/ethnic minority populations. Health Promot Pract. 2021;22(5):714-723. doi:10.1177/1524839920933897 32552138

[19] Heinert S, Escobar-Schulz S, Jackson M, . Barriers and facilitators to hypertension control following participation in a church-based hypertension intervention study. Am J Health Promot. 2020;34(1):52-58. doi:10.1177/0890117119868384 31409096

[20] Carter BL, Bosworth HB, Green BB. The hypertension team: the role of the pharmacist, nurse, and teamwork in hypertension therapy. J Clin Hypertens (Greenwich). 2012;14(1):51-65. doi:10.1111/j.1751-7176.2011.00542.x 22235824 PMC3257828

[21] Prendergast HM, Petzel-Gimbar R, Kitsiou S, . Targeting of uncontrolled hypertension in the emergency department (TOUCHED): design of a randomized controlled trial. Contemp Clin Trials. 2021;102:106283. doi:10.1016/j.cct.2021.106283 33484897 PMC8272286

[22] Chobanian AV, Bakris GL, Black HR, ; National Heart, Lung, and Blood Institute Joint National Committee on Prevention, Detection, Evaluation, and Treatment of High Blood Pressure; National High Blood Pressure Education Program Coordinating Committee. The Seventh report of the Joint National Committee on Prevention, Detection, Evaluation, and Treatment of High Blood Pressure: the JNC 7 report. JAMA. 2003;289(19):2560-2572. doi:10.1001/jama.289.19.2560 12748199

[23] Schulz KF, Altman DG, Moher D; CONSORT Group. CONSORT 2010 statement: updated guidelines for reporting parallel group randomized trials. Obstet Gynecol. 2010;115(5):1063-1070. doi:10.1097/AOG.0b013e3181d9d421 20410783

[24] Kitsiou S, Thomas M, Marai GE, . Development of an innovative mhealth platform for remote physical activity monitoring and health coaching of cardiac rehabilitation patients. Paper presented at: IEEE EMBS International Conference on Biomedical and Health Informatics; February 16, 2017; Orlando, FL.

[25] Frison L, Pocock SJ. Repeated measures in clinical trials: analysis using mean summary statistics and its implications for design. Stat Med. 1992;11(13):1685-1704. doi:10.1002/sim.4780111304 1485053

[26] Kieser M, Friede T. Simple procedures for blinded sample size adjustment that do not affect the type I error rate. Stat Med. 2003;22(23):3571-3581. doi:10.1002/sim.1585 14652861

[27] Proia KK, Thota AB, Njie GJ, ; Community Preventive Services Task Force. Team-based care and improved blood pressure control: a community guide systematic review. Am J Prev Med. 2014;47(1):86-99. doi:10.1016/j.amepre.2014.03.004 24933494 PMC4672378

[28] Anderegg MD, Gums TH, Uribe L, Coffey CS, James PA, Carter BL. Physician–pharmacist collaborative management: narrowing the socioeconomic blood pressure gap. Hypertension. 2016;68(5):1314-1320. doi:10.1161/HYPERTENSIONAHA.116.08043 27600181 PMC5063695

[29] Community Preventive Services Task Force. Team-based care to improve blood pressure control: recommendation of the Community Preventive Services Task Force. Am J Prev Med. 2014;47(1):100-102. doi:10.1016/j.amepre.2014.03.003 24933493

[30] Carnethon MR, Pu J, Howard G, ; American Heart Association Council on Epidemiology and Prevention; Council on Cardiovascular Disease in the Young; Council on Cardiovascular and Stroke Nursing; Council on Clinical Cardiology; Council on Functional Genomics and Translational Biology; and Stroke Council. Cardiovascular health in African Americans: a scientific statement from the American Heart Association. Circulation. 2017;136(21):e393-e423. doi:10.1161/CIR.0000000000000534 29061565

[31] Caraballo C, Massey DS, Ndumele CD, . Excess mortality and years of potential life lost among the Black population in the US, 1999-2020. JAMA. 2023;329(19):1662-1670. doi:10.1001/jama.2023.7022 37191702 PMC10189563

[32] Keenan NL, Rosendorf KA; Centers for Disease Control and Prevention (CDC). Prevalence of hypertension and controlled hypertension—US, 2005-2008. MMWR Suppl. 2011;60(1)(suppl):94-97. 21430632

[33] Antonakoudis G, Poulimenos L, Kifnidis K, Zouras C, Antonakoudis H. Blood pressure control and cardiovascular risk reduction. Hippokratia. 2007;11(3):114-119. 19582204 PMC2658793

[34] Pearson ML, Mattke S, Shaw R, Ridgely MS, Wiseman SH. Patient self-management support programs: an evaluation. Accessed March 19, 2025. https://www.ahrq.gov/sites/default/files/publications/files/ptmgmt.pdf

[35] Bress AP, Anderson TS, Flack JM, ; American Heart Association Council on Hypertension; Council on Cardiovascular and Stroke Nursing; and Council on Clinical Cardiology. The management of elevated blood pressure in the acute care setting: a scientific statement from the American Heart Association. Hypertension. 2024;81(8):e94-e106. doi:10.1161/HYP.0000000000000238 38804130

[36] Ma MA, Gutiérrez DE, Frausto JM, Al-Delaimy WK. Minority representation in clinical trials in the US: trends over the past 25 years. Mayo Clin Proc. 2021;96(1):264-266. doi:10.1016/j.mayocp.2020.10.027 33413830

