# Supplementary material for: Emergency Department–Based Education and mHealth Empowerment Intervention for Hypertension: The TOUCHED Randomized Clinical Trial
Source: JAMA Cardiol. 2025 Apr 23;10(7):657–65. doi: 10.1001/jamacardio.2025.0675 (PMC12019670; doi:10.1001/jamacardio.2025.0675)
Supplement: Supplement 1. — Trial Protocol. [file jamacardiol-e250675-s001.pdf]

Targeting of Uncontrolled Hypertension in the Emergency Department (TOUCHED)

Principal Investigator:

Heather M. Prendergast MD, MS, MPH, Professor

Department of Emergency Medicine

Co-Investigators:

Renee Petzel Gimbar, PharmD

Spyros Kitsiou, PhD

Martha Daviglus, MD, PhD

Sally Freels, PhD

Study Location(s): University of Illinois Emergency Department

1740 W. Taylor, Chicago, Il 60612

Sponsor: National Institutes of Health (NIH)

Version 1.8, Date: May 10, 2022

1.0 PROJECT SUMMARY/ABSTRACT

Effective interventions that can address uncontrolled hypertension, particularly in underrepresented populations that use the emergency department (**ED**) for primary care, are critically needed.[1, 2] [3] **Uncontrolled hypertension (HTN)** contributes significantly to cardiovascular morbidity and mortality and **is more frequently encountered among patients presenting to the ED.** [4, 5] **EDs serve as the point of entry into the health care system for many high-risk patient populations, particularly minority and low-income individuals.** Preliminary data from our research group, based on a largely minority patient population presenting to the ED, demonstrated significant rates of subclinical heart disease (diastolic dysfunction and left ventricular hypertrophy) in those with elevated blood pressures.[6] These early echocardiogram changes are reversible with existing strategies to improve blood pressure control making ED interventions within this population imperative. [7] [8]

The proposed project underscores the following: **1)** The prevalence of uncontrolled/undiagnosed HTN in underrepresented groups presenting to the ED is alarmingly high, and **2)** ED engagement and early risk assessment/stratification is a cost-effective, feasible innovation to help close health disparity gaps in HTN. This proposal involves a ***two-arm randomized controlled trial of up to 770 patients from the Emergency Department at University of Illinois Hospital with elevated blood pressure (BP). The primary objective is to determine the effectiveness of an emergency department-initiated Educational and Empowerment (*** E^2 ***) intervention with a Post Acute Care Hypertension Consultation (PACHT-c) intervention (arm 2) on the primary outcome of mean systolic blood pressure (SBP) differences between the two trial arms at 6-months post intervention. Secondary objectives include evaluating the effectiveness of this ED education and empowerment intervention on mean SBP and diastolic blood pressure (DBP) differences at 3-months, and mean DBP differences at 6-months. Additionally improvement in cardiovascular risk score (as measured by the Atherosclerotic Cardiovascular Disease (ASCVD) Risk Score), medication adherence (as measured by the Modified Morisky Scale), and primary care engagement (measured by compliance with outpatient follow-up appointments), will also be assessed as part of this study.***

2.0 BACKGROUND/SCIENTIFIC RATIONALE

A. Background - EDs serve a high-risk population that is not readily captured in other clinical settings with many patients utilizing the ED as part of their primary care access. [3, 9] Moreover, patients who report using the ED as their usual site of care are disproportionately more likely to have poor cardiovascular outcomes relative to private doctor's offices [10]

Why Study Uncontrolled Hypertension in the Emergency Department Setting? It is estimated that the prevalence of uncontrolled/undiagnosed hypertension in the ED is as high as 45%. [11] Hypertensive patients presenting to the ED are a particularly high-risk group with >50% having stage II or higher hypertension (SBP > 160 or DBP >100).[4] Many cases of elevated blood pressures in the ED are incidental findings and not related to the patient's chief complaints. Incidental hypertension represents a quandary for the emergency physician who cannot guarantee follow-up treatment.[12, 13] Thus identification and referral rates remain low in patients presenting to the ED with moderate BP elevations despite published outpatient guidelines. A recent study reported approximately 59% of men and 78% of women with elevated BP in the ED had continued elevated readings at outpatient follow-up after the ED visit. [14] Several studies have found that only 7-25% of ED patients with elevated BP are given instructions for outpatient BP follow-up. [12-14] Currently, **there is no risk assessment or stratification based upon blood pressure assessment performed on hypertensive patients prior to discharge from the ED**. All patients receive the same standard discharge instructions from the ED to follow up with a primary care provider (PCP). Recent emergency medicine literature suggests that if evidence-based guidelines were available for management of these patients and more assured follow-up mechanisms were in place, there would be greater compliance with referral guidelines and heightened awareness of secondary prevention interventions available to ED physicians. [11]

In 2010, there were 128 million ED visits, and by 2012, this number had increased to 138 million. [15] The proposed study is novel because it is initiated in the emergency department, is an innovative change to the current care delivery model, utilizes mobile health home BP monitoring, and can decrease the health disparity gaps associated with uncontrolled HTN. EDs

serve as the point of entry into the health care system for many high-risk patient populations, particularly minority and low-income individuals, who are not readily captured in other clinical settings because a significant number of these patients do not have a medical home and routinely utilize the ED as their source of primary care. EDs are well suited at the interface between inpatient and outpatient care and can contribute to reducing the health disparity associated with uncontrolled HTN in high-risk minority populations through focused engagement.

3.0 OBJECTIVE/AIMS

The specific aims and hypotheses to be tested are:

Aim 1: Evaluate the effectiveness of an ED-based E^2 + PACHT-c intervention (arm 2) on the primary outcome *of mean SBP difference* at 6-months post-intervention compared to usual care (arm 1).

H1: The mean SBP difference (from baseline) will be significantly greater in the E^2+ PACHT-c group (arm 2) compared to the usual care group (arm 1) at 6- months post-randomization, i.e., SBP change in arm 2 > arm 1 at 6-months post-randomization.

Aim 2: Evaluate the effectiveness of an ED-based E2 intervention with PACHT-c on the secondary outcome of mean SBP and DBP differences at 3-months and 12-months, and mean DBP differences at 6 months post-intervention compared to usual care.

H2: The mean SBP and DBP differences from baseline to 3- months and baseline to 12-months post-intervention and mean DBP at 6-months post intervention will be significantly greater in the ED-based E2 intervention compared to the usual care group, i.e., SBP and DBP change in arm 2 > arm 1 at 3 –months and 12-months post-intervention and DBP change in arm 2 > arm 1 at 6 -months post-intervention.

Aim 3: Examine if the E2 intervention reduces racial disparities. Specifically, if the intervention is as effective in racial minorities as non-minorities. This will be an exploratory analysis and will primarily focus on changes in cardiovascular risk score/profile. Also included will be an assessment of intervention mediators: primary care engagement, medication adherence, and HTN knowledge in these same patients at 3 and 6 -months post randomization-intervention.

4.0 ELIGIBILITY

The UI Health Department of Emergency Medicine serves a diverse population in Chicago and is a Level II trauma center located within the Illinois Medical District.

All patient recruitment will be completed in the UI Health Department of Emergency Medicine (ED). ED study personnel will approach patients who meet inclusion criteria for the study. Study personnel will be available on rotating shifts (including days, nights, and weekends) in order to facilitate ongoing patient recruitment and enrollment. Individuals deemed eligible will be consented and sign a written consent with HIPAA authorization and have baseline data collection. The research assistants (RAs) will inform patients that the research is being conducted to improve hypertension knowledge and blood pressure control by facilitating primary care connections and to determine if the ED can help people reach goals of treatment.

4.1 Inclusion Criteria:

Inclusion criteria: Stage 1 Hypertension- Elevated Blood Pressure of $\geq$140/90 and $\leq$ 180/110 at time of discharge from ED; Verbal fluency in English or Spanish; Age 18 to 75 years.

4.2 Exclusion Criteria:

Exclusion criteria: Unable to verbalize comprehension of study or impaired decision making or documented dementia; Lives outside Chicago communities or plans to move from Chicago area within the next year; Pregnant or trying to get pregnant; COVID-19 positive.

4.3 Vulnerable Populations:

Study participants will be patients with uncontrolled/undiagnosed HTN in underrepresented groups presenting to the emergency department. ED engagement and early risk assessment/stratification is aimed to be a cost-effective, feasible, and minimally invasive innovation to help close health disparity gaps in HTN.

Collaborating Sites:

There are no collaborating sites.

5.0 SUBJECT ENROLLMENT

In order to minimize the possibility of coercion or undue influence on potential subjects, subjects will only be approached for study consent after they have completed their evaluation in the emergency department and have been identified for discharge. Subjects will also be informed that participation is voluntary and will not affect their relationship with the university/hospital if they decline to participate. Also, patients that are being cared for by the PI or key personnel will not be approached and consented by that individual but rather another member of the study team . Subjects will initially be identified by review of the tracking board listing in the department. The tracking board displays the patients' last name, room number, age, and vital signs. If patients meet the BP requirement (BP ≥ 140/90 and ≤180/110) and are slated for discharge from the emergency department, then a clinical EMT will approach the attending physician caring for the patient for preliminary review of exclusion criteria. Eligible participants will be approached by the clinical EMT with an iPad. The research assistant will communicate with the potential participant via the iPad. The consent form will be electronic to avoid possible contamination. Additionally, participants will be informed that a thank you letter will be mailed to their home after completion of the study. At no time will any information about refusals to participate be placed in the subjects' medical record. All data will be entered into electronic database (REDCap). There will be no paper charts/data entry. This will minimize the potential for loss of information and breech of privacy. All of this information will be handled in accordance with HIPAA guidelines. We do not expect subjects to experience discomfort with the collection of the minimally invasive clinical measures collected.

6.0 STUDY DESIGN AND PROCEDURES

Human Subjects Involvement: The proposed study is a randomized, controlled trial focused on a high-risk ED population with evidence of moderately elevated blood pressures (≥140/90 and ≤180/110). 770 ED patients (predominately African-American and Latino patients, based upon the demographics of the UI Health patient population) will be randomized to one of two arms: 1.) usual care (preprinted discharge instructions and a 48-72 hour referral to our FQHC program or assigned provider as appropriate) or 2) the ED-initiated Educational and Empowerment (E^2) intervention and PACHTc intervention followed by 48-72 hour referral to our FQHC (or assigned health center). There will be 385 subjects in each arm.

Figure 1. Study Design

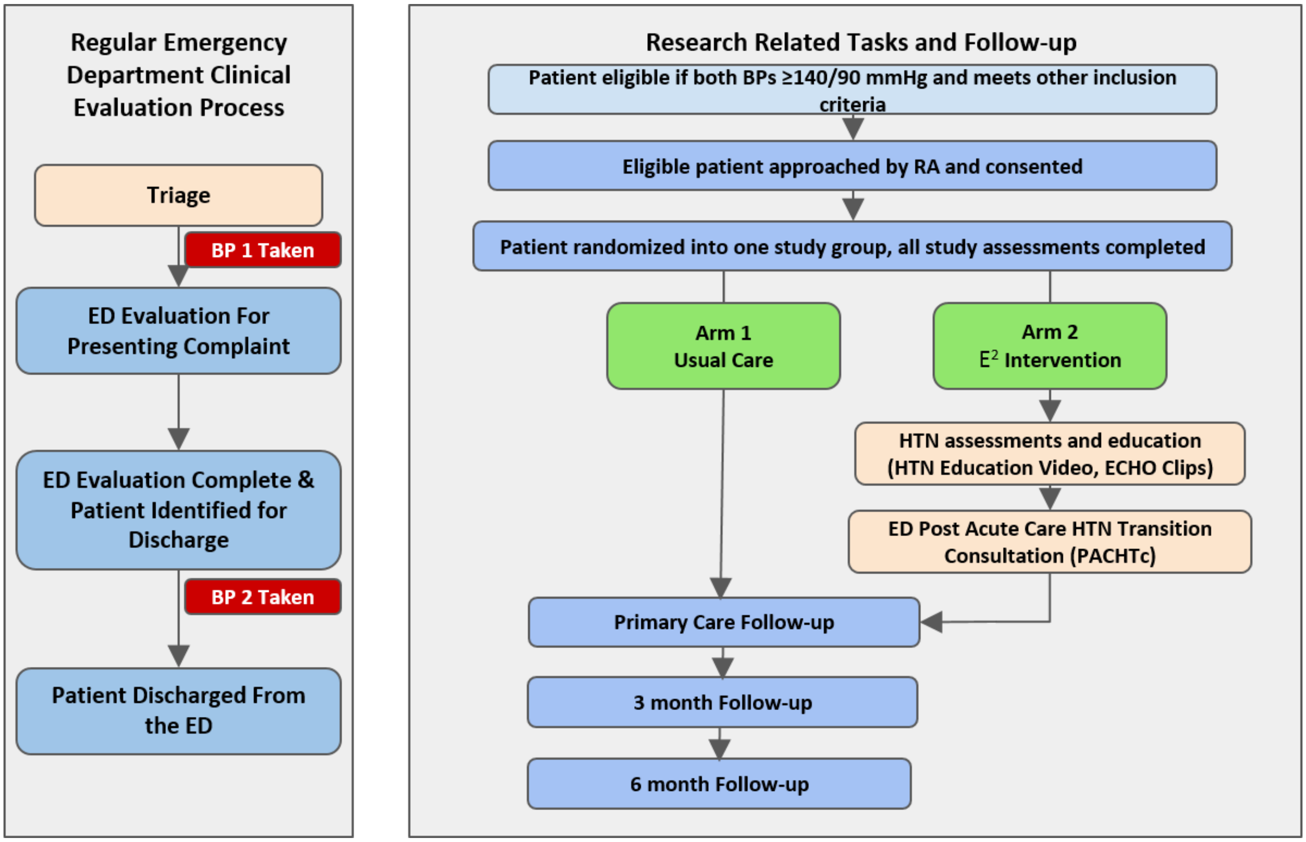

The E^2 intervention (arm 2) consists of a series of risk assessment tools (surveys, video, and minimally invasive bedside assessments) designed to be efficient, patient-centered and educational for participants in an emergency department setting. Through the intervention, participants will learn more about hypertension management and secondary complications associated with uncontrolled BP, such as subclinical heart disease.

Subclinical heart disease refers to the presence of early echocardiogram changes seen on limited beside echocardiograms often as a result of uncontrolled hypertension. These changes include diastolic dysfunction and left ventricular hypertrophy. Many of these changes can be reversible with hypertension control. Study participants will not be diagnosed with subclinical heart disease in the ED. Instead participants will be shown limited bedside echocardiogram video clips of gender matched echocardiograms with subclinical changes as a part of an empowerment tool of the study.

Using Atherosclerotic Cardiovascular Disease (ASCVD) risk score calculators preloaded on study iPads, participants will have their risk score calculated by entering their age, tobacco status (Y/N), Systolic BP, Cholesterol/HDL, and BP treatment (Y/N). Point of care cholesterol/HDL measurement will be obtained using the Polymer Technology Systems CardioCheck PA Analyzer, which analyzes up to 4 cholesterol types with a single fingerstick of blood and is CLIA-waived and FDA approved. Trained nurse practitioners and emergency medical technicians will be performing the cholesterol tests.

Additionally, all participants in arm 2 will receive a Post-Acute Care Hypertension Transition Consultation (PACHTC) with a clinical pharmacist or APN. To ensure consistency throughout the trial, the PACHTC will be standardized and interchangeable between the clinical pharmacists and APNs. During the day, the on-site ED clinical pharmacist will provide consultations. During evening and weekend hours, consultations will be provided by the APN located in the ED Clinical Decision Unit (CDU).

Finally, all participants randomized to arm 2 will receive an FDA-approved HBPM kit that includes the Nokia wireless (self-inflating) BPM monitor and Health Mate mobile app. Participants without a mobile device will be provided one for the study.

The app automatically launches when the patient slips on the cuff and turns on the monitor to measure his/her BP. All BP readings automatically sync with the app, which creates an easy-to-understand chart of all the measurements and provides participants with instant color-coded feedback based on AHA recommendations for hypertension. Synced data are automatically uploaded from the mobile app to the iCardia server of our study.

Participants will be shown how to use the BP monitor and app by a clinical EMT, view a standardized 2-minute instructional video, and asked to confirm/demonstrate that they understood how to use the device and app using the teach-back method prior to leaving the ED. Participants will be asked to check their BP at home, preferably daily but a minimum of twice per month, and use the mobile app to view their data. The EMT will set-up daily or bi-weekly automatic reminders in each participant's mobile app that will prompt them to measure their BP at a preferred time.

In case of missed measurements for 1 consecutive week, participants will receive one text-message per day through the iCardia platform for four consecutive days until they complete a BP measurement. In case of missed measurements for 14 consecutive days, participants will be contacted by phone by the RA's. All data will be remotely monitored by the RA's through the iCardia platform. The patient-centered benefit of HBPM monitoring has been repeatedly shown and is recommended as an important component of HTN management. HBPM has been shown to improve adherence to medications, induce healthy lifestyle changes, and aid in optimizing of treatment. In addition, use of the HBPM values will serve as a safety feature to avoid pushing BP too low in study participants.

Additionally, participants will be sent behavioral change text messages. The text messages come from a pool of validated messages chosen by the investigators. They promote medication adherence and BP measurement adherence. The message database will be submitted along with this document. Participants will receive 3 behavioral text messages per week at a time of their preference, in addition to the app notifications they receive each day to monitor their blood pressure. Text messages will be programmed in iCardia by research staff.

The Usual Care group (Arm 1) will received preprinted discharge instructions and an outpatient referral. This group represents standard of care. All other interventions in arm 2 are research activities.

Table 1: Intervention Components by Arm *

**Arm 1: Usual Care (N=385)**<br>**Arm 2: E^2 Intervention (N=385)**				
**Intervention Components by Arm**	Baseline	Quarterly	Mid-point	End
Location	ED	Phone	UI-Health Suite 1600	UI-Health Suite 1600
Time Point in Months (0=ED discharge)	0	1, 3, 5	3	6
Duration	10-20 min	5 min	10-20 min	20 min
BP Measured	1, 2		1, 2	1, 2
Clinical Data (from med chart)	1, 2			
Atherosclerotic Cardiovascular Disease	1,2			1,2

(ASCVD) Risk Score				
Hypertension Knowledge Assessment	1, 2			1, 2
Modified Morisky Scale	1, 2	1, 2	1, 2	1, 2
Patient Activation Measurements Survey (PAM)	1, 2	1, 2	1, 2	1, 2
Verify patient contact info	1, 2	1, 2	1, 2	
Bedside Echocardiogram Education	2			
Hypertension Education Video	2			
PACT-Hypertension (Pharmacy) Consultation:	2			
Home BP Monitoring Training and Reminders	2	2	2	

*We may ask participants to return for a follow up at one year post-enrollment at which time all assessments and surveys performed at recruitment will be repeated.

<u>Details of ED Screening (risk assessment, both arms)</u>

BP Measurement: Prior to randomization all participants will be screened using a standardized 5- point pain scale with 5 indicating severe pain and 0 no pain. Participants with moderate to severe pain (3-5) will not be randomized until their pain scale is improved (0-2). Participants will then have a standard BP measurement taken (as per AHA guidelines) to ensure consistency and standardization of BP measurements throughout the trial.

The blood pressure measurements will be obtained using standard monitors currently available in our Emergency Department: Welch Allyn, Model VSM 6000 series, Configuration Number: NIBP, Pulse Rate, SPO2, Temp, Hardware Version P3, Software Version 1.71.03.

The blood pressure measurement protocol based on the AHA guidelines are as follow:

1. The patient will be asked to remove all clothing that covers the location of cuff placement.

2. The individual will be comfortably seated, with the legs uncrossed, and the back and arm supported, such that the middle of the cuff on the upper arm is at the level of the right atrium (the mid-point of the sternum).
3. The patient will be instructed to relax as much as possible and to not talk during the measurement procedure. The patient will be asked to sit quietly for 5 minutes before the first reading is taken.
4. A minimum of three readings will be taken and the average of the last two readings will be used to record the measurement. There will be intervals of at least 1 minute between readings.
5. If there is >4 mm Hg difference between the second and third readings, additional (one or two) readings will be obtained and then the average of the two closest readings will be used.

Atherosclerotic Cardiovascular Disease (ASCVD) Risk Score: Using ASCVD Risk Score calculators preloaded on study iPads, participants will have their risk score calculated by entering their age, tobacco status (Y/N), Systolic BP, Cholesterol/HDL, and BP treatment (Y/N). Point of care cholesterol/HDL measurement will be obtained using the Polymer Technology Systems CardioChek PA Analyzer, which analyzes up to 4 cholesterol types with a single fingerstick of blood and is CLIA-waived and FDA approved.

Hypertension Knowledge Survey (all arms): The hypertension knowledge survey is a 10-item, validated tool developed to assess hypertension knowledge in low literacy patient populations. The scale assesses respondents' knowledge in defining hypertension, lifestyle, and behaviors that may affect BP levels, and the long-term consequences of HTN. The survey has been validated in an urban population that included a high proportion of black and Latino patients. Scores are categorized into tertiles that indicate low (≤7), medium (8), or high (9-10) levels of HTN knowledge.

Modified Morisky Scale Health Survey and Patient Activation Measurement Survey (PAM) (all arms): The modified Morisky scale is a validated 4-item instrument to assess self-reported patient adherence related to antihypertensive medication. The modified Morisky scale provides a total score with a range of 0 to 4, with higher scores indicating lower adherence to medication. The scores of the modified Morisky scale can be classified as low compliers (3-4), medium

compliers (1-2) and high compliers (0) based on its criterion validity with BP control. The Patient Activation Measurement Survey (PAM) is a 10-item instrument that measures level of engagement (activation) and has been used and validated in HTN interventions with higher scores meaning better self-care behaviors. The surveys will be administered by RAs at the time of enrollment and at 3, 6, and 12 months during the in-person visit.

<u>Details of Intervention Components</u>

HTN Educational Video. The video will educate participants in arm 2 about high BP, how it is diagnosed, and the importance of treating it to prevent secondary complications. Based upon the existing literature overall HTN knowledge is extremely low among minority populations. Participants will review a 3-5 minute video on HTN, The customized video will be bi-lingual and culturally sensitive. After the video participants will complete a short touch-screen self-assessment with real-time computerized feedback.

Visual Echocardiogram Image Clips: We are using visual images of gender-matched echocardiograms as a tool to educate and motivate patients to change their behavior to improve their BP. We have found that **the real time visualization of cardiac ultrasound images with active discussion of findings is a significant patient motivator and empowerment tool,** and was a significant factor in the success of a previous pilot study. (Figure 2)

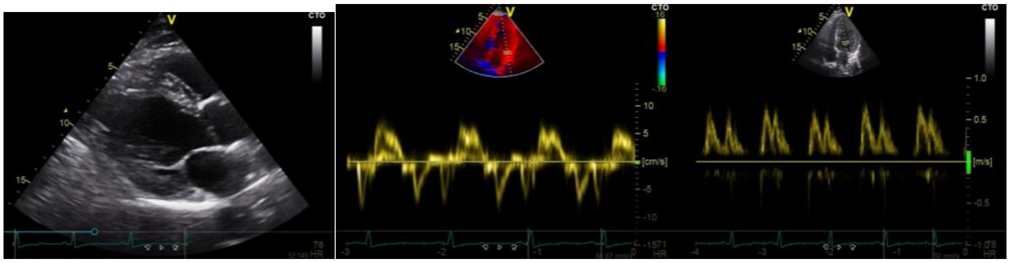

Figure 2. Images of a patient with mild to moderate diastolic dysfunction as evidenced by a normal E/A ratio, but an e' lateral mitral valve annular velocity < 10 cm/s. Patients are better informed about consequences of untreated HTN when they can visualize the effects of HTN on the heart.

Smartphone-enabled BP monitoring Kit: All participants randomized to the intervention group will receive an FDA-approved home BP monitoring kit that includes the Nokia wireless (self-inflating) BPM monitor and Health Mate mobile app. The app automatically launches when the

patient slips on the cuff and turns on the monitor to measure his/her BP. All BP readings automatically sync with the app, which creates an easy-to-understand chart of all the measurements and provides participants with instant color-coded feedback based on AHA recommendations for hypertension. Synced data are automatically uploaded from the mobile app to the iCardia server of our study. Participants will be shown how to use the BP monitor and app by RAs, view a standardized 2-minute instructional video and asked to confirm/demonstrate that they understood how to use the device and app using the teach-back method prior to leaving the ED. Participants will be asked to check their BP at home daily and use the mobile app to view their data. The EMT's will set-up daily automatic reminders in each participant's mobile app that will prompt them to measure their BP at a preferred time. In case of missed measurements for three consecutive days, participants will receive one text-message per day through the iCardia platform for four consecutive days until they complete a BP measurement. In case of missed measurements for 7 consecutive days, participants will be contacted by phone by the RA's. Participants will also receive 3 behavioral text messages per week that are aimed at improving medication adherence and BP measurement adherence. All data will be remotely monitored by the RA's through the iCardia platform. Participants will be informed that the Health Mate app is not a telemonitoring app and in the event the participant is experiencing a hypertensive crisis (>180/120) they must contact a physician immediately. The patient-centered benefit of home BP monitoring has been repeatedly shown and is recommended as an important component of HTN management. Home BP monitoring has been shown to improve adherence to medications, induce healthy lifestyle changes, and aid in optimizing of treatment. In addition, use of the home BP monitoring values will serve as a safety feature to avoid pushing BP too low in study participants.

PACHT-c: All participants randomized to the E^2 intervention will have a focused consultation with either a clinical pharmacist or an APN. To ensure consistency throughout the trial, the PACHT-c intervention will be standardized and interchangeable between the clinical pharmacists and APNs. During the day, the on-site ED clinical pharmacist will provide consultations. During evening and weekend hours, consultations will be provided by the APN located in the ED Clinical Decision Unit (CDU). The CDU is open 24 hours a day, 7 days a week. During this consultation, the pharmacist/APN repeats the BP measurement; reviews the screening

assessments; and reviews general principles of BP control including nutrition, exercise, and smoking cessation. BP will be managed according to the current published guidelines available regarding initiation of first-line antihypertensive medications. Patients with BP ≥140/90 mmHg may be started on antihypertensive medications by the provider during the consultation if appropriate. All PACHT-c clinic notes will be accessible in the EMR, which is shared between both the ED and our FQHC, Mile Square Health Center. No tests will be ordered during the pharmacy follow-up.

Based on our pilot trial (IRB Protocol # 2015-0323) the E^2 intervention takes about 20-30 minutes following randomization. Once patients are identified for discharge, the wait time before physically leaving the ED provides ample time to consent and complete the initial assessments based on participant randomization at the bedside without adding delays. Participants will be approached for study participation after they have been identified for discharge and BEFORE being physically discharged from the ED.

<u>Details of Additional Program Components:</u>

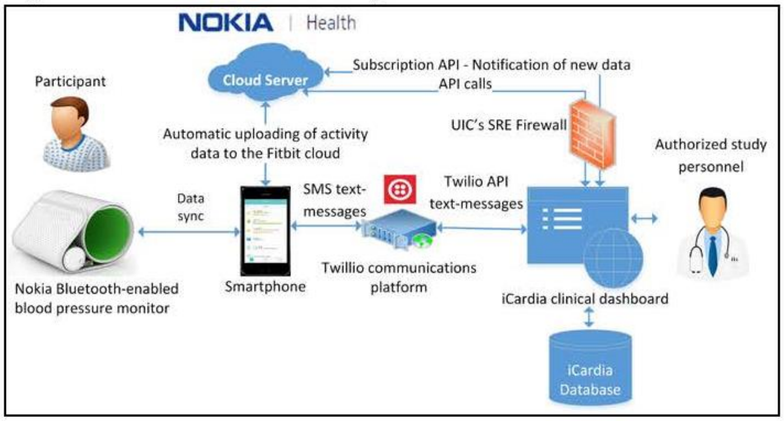

iCardia & Remote BP Monitoring: iCardia is a secure password-protected remote monitoring system hosted in a HIPAA-compliant server at UI Health. iCardia provides a user-friendly environment for authorized personnel to view participants' BP readings in real-time in the form of graphs and send personalized text-messages to participants' cell phones (as needed) through the Twilio communication platform (**Figure 3**). RAs will receive training on iCardia and schedule messages for study participants regarding appointments, medications, and goals. Text messages can be sent immediately, recurrently (e.g. daily), or at a scheduled date/time to one or more participants. All communications utilize Transport Layer Security with encryption.

Addressing "White Coat HTN": It has been suggested that a "white coat effect" (increase in BP

primarily in the medical care environment) can be present in as many as 20-33% of patients diagnosed with HTN. The use of wireless BP monitors with remote monitoring will address this issue and ensure that this is not a significant confounder in our study population. Following enrollment, subjects will be asked to check their BPs daily.

Study Coordinator: The study coordinator will be responsible for overseeing the 48-72 hour HTN outpatient referrals for all study participants and track compliance with follow-up appointments using a combination of EMR review and participant self-report. The study coordinator will also assist with transportation concerns, fax PACHT-c letters to non-UI Health providers, and be trained to use the Open Access scheduling system for selected FQHCs to facilitate appointment scheduling.

Illinois Video Interpreter Network (IVIN) (all arms): Translators versed in healthcare terminology are available by video 24 hours a day. In the rare circumstance that a Spanish-speaking study staff member is not available, the IVIN system will be employed to facilitate communication with the participant both at enrollment in the ED and during the PACHT-consultation.

Patient In-Person Clinic Follow Ups (all arms): Following enrollment and randomization, **there will be up to three additional in-person visits at 3 and 6-months for BP measurements**. Staff will be blinded to study arm assignment. These repeat visits will occur in the PACTH-c space (adjacent to the ED in Suite 1100 of the hospital) and be conducted by trained RAs using study protocols and the AHA guidelines.

<u>Participant Retention (all arms):</u> Attrition is a challenge for follow-up data collection, particularly in underserved populations. We plan to use several strategies including: **1**) monetary reimbursement for in-person data collection ($50 for their 3 month follow up and $50 for their 6 month follow-up post-randomization); $50 will also be given to participants in the event they are asked to return in 1 year); $100 for participants that miss their 3 month follow-up but come in for their 6 month follow-up ($50 for missed 3 month and $50 for 6 month which is main outcome); **2**) periodic phone calls to verify address and phone data (at 1, 3, 5, 6, 9, and 11 -months post-enrollment); **3**) use of secondary alternative contact information; **4**) text messaging 24 hours prior to follow-up appointments; **5**) mailing birthday/holiday cards; and **6**) monthly newsletters

containing recruitment updates. Co-Investigator Daviglus has demonstrated success in participant retention in numerous studies involving high-risk populations with attrition rates between 8-10%. In our R56 trial (IRB protocol # 2015-0323), we had a final attrition rate of 25% (9-month follow-up) due to transportation difficulties and loss of phone contact. To limit the attrition rate for this proposal, we will provide ride share, public transit, and parking vouchers as needed, and will provide smartphones to Arm 2 study participants without phones. Geocoding of our patient population demonstrated that the majority of individuals live within a 5-10-mile radius of UI Health. We are confident that these retention strategies will limit our attrition rate to no more than 20%.

Assessment of Impact of Intervention on the Racial Disparity Associated with Uncontrolled HTN and Mediating Factors (Aim 3)**:** We will examine whether the intervention reduces racial disparities associated with uncontrolled HTN, i.e. is this intervention as effective in ethnic minorities as non-minorities. This will be an exploratory analysis and will primarily focus on changes in cardiovascular risk score. In addition, we will measure two potential mediating factors multiple times during the trial: **1)** adherence to follow-up visits, and **2)** the Modified Morisky Scale. All participants will receive phone calls at predetermined times to determine **primary care engagement**. All communication will be scripted and include: **1)** whether follow-up visits with the PCP have occurred or are scheduled; and **2)** the Modified Morisky Scale to assess current HTN medication use and understanding. The appropriate surveys will be administered by RAs at the time of enrollment, by phone at 1 and 5 months, and at 3 and 6 months during the in-person visit. Shorter calls will be made at 2, 5, and months to schedule the 3 and 6-month appointments.

Research Material:

All research materials obtained during the course of this study will be for research purposes. The materials that will be obtained from the human subjects that participate in this study are blood pressure measurements, as well as assessments on medication adherence, HTN knowledge, and patient activation.

New Data:

Demographic data and blood pressure data, both provider-obtained and/or from patient-recorded home measures, will be obtained for all participants. New data will also include level of medication adherence, hypertension knowledge, and ASCVD risk score.

Linkages to Subjects:

All information collected in this study is confidential and all data will be transformed into anonymously-coded identifiers for each subject prior to analysis. We use a double coding system to maintain the confidentiality of information. Only Dr. Prendergast (PI) and study personnel will be able to link information to subjects. This is necessary because patients will be followed for a total of six months after discharge so participants will need to be tracked during this time. All data will be stored electronically on REDCap.

For Specimen Collection Studies

We will collect a single fingerstick of blood to measure HDL cholesterol levels for the ASCVD risk score.

For Studies that Collect Existing or Prospective Data

Prospective data to be collected from all subjects include blood pressure measurements. Subjects in the E^2 intervention will have a series of risk assessment tools designed to be efficient, patient-centered and educational for participants in an emergency department setting. Through the intervention, participants will learn more about hypertension management and complications associated with uncontrolled BP.

Study data will be maintained for 7 years post study completion and then will be destroyed.

7.0 EXPECTED RISKS/BENEFITS

Potential Risks:

The potential risks associated with this study are: (1) The potential for loss of privacy or confidentiality of health information. (2) Possible discomfort with assessments, however all measures are noninvasive with the exception of a single fingerstick of blood for the ASCVD risk score, so little discomfort is expected. (3) For the group that receives the Smartphone-enabled

Blood Pressure Monitoring Kit (group 2), participants may potentially feel annoyed or irritated with the frequency of notifications and/or text-message reminders they receive. For the app notifications, participants will have the option of turning off the notifications or change their frequency. The Research Assistants will show participants how to do this when they set up the app on the participant's phone. For the text-messages, participants will have two options; the first option is to contact the research staff and ask them to reduce the number of text-messages they receive. The second option is to reply to one of the text-messages with the word "STOP". Our system will automatically stop sending text-messages after that. However, if participants want to re-subscribe, they can reply with the word "START" and the text-messaging program will resume.

Alternative Treatments/Procedures:

Patients that decide not to enter this study will be informed that there is other care available, such as a referral to see a primary care doctor at the Mile Square Health Center. The study research assistant will discuss these options with the patient and let them know that they do not have to be in this study to be treated for hypertension.

Potential Benefits of Proposed Research to Subjects and Others:

Subjects may not directly benefit from participation in the research. Indirect benefits include expansion of risk assessment intervention to include emergency departments.

Importance of the Knowledge to be Gained:

Uncontrolled hypertension contributes significantly to cardiovascular morbidity and mortality and is more frequently encountered among patients presenting to the emergency department (ED), which serves as the point of entry into the health care system for many high-risk patient populations, particularly minorities. If our results are as expected, our clinically relevant study has the potential to provide new insights into increasing motivation, follow-up rates, and consequently, treatment compliance and blood pressure control in a predominately underserved hypertensive population.

8.0 DATA COLLECTION AND MANAGEMENT PROCEDURES

Data Characteristics:

All data forms will be initially developed and tested on paper; Spanish language versions of participant questionnaires will be prepared and back-translated to English prior to obtaining certified translations; after thorough pilot testing, paper forms are to be converted to equivalent electronic data entry forms with use of REDCap. REDCap (Research Electronic Data Capture) is a secure, web-based application designed to support data capture for research studies, providing: 1) an intuitive interface for validated data entry; 2) audit trails for tracking data manipulation and export procedures; 3) automated export procedures for seamless data downloads to common statistical packages; and 4) procedures for importing data from external sources. At UI Health, REDCap is hosted on secure servers located in and supported by the Institute for Health Research and Policy (IHRP). Project staff will be trained in the use of REDCap for entry of study data. Appropriate logic and limits checking (including cross-form validity checks) will be implemented in REDCap to facilitate accurate and consistent data entry. Where practical, study data will be directly entered into the REDCap database by clinical staff. Alternatively, information can be captured on paper and entered into the system from the forms. Routinely throughout the study the REDCap database will be downloaded into a secure virtual computing environment (virtual servers and virtual workstations secured behind software and hardware firewalls) available through the IHRP. Study information within the virtual environment will be available for participant tracking activities such as production of result and reminder letters, scheduling of interim telephone contacts, and scheduling and tracking of follow-up visits. Regular quality control and data cleaning/ error reports will be produced which identify missing, out of range, and questionable values. These reports will be reviewed with study staff every two weeks. Errors will remain on reports until they are resolved. Statistical analyses prior to the 3, 6, and 12- month outcome evaluations will be limited to quality control, enrollment and drop out reporting and participant demographics. No interim looks are anticipated but quality control and data entry errors and omissions will be continuously monitored and reported to staff for remediation.

9.0 DATA ANALYSIS

Data Analysis will be performed by Dr. Ramon Durazo-Arvizu at the Institute for Minority Health Research at the University of Illinois Chicago and Dr. Sally Freels.

10.0/11.0 QUALITY CONTROL AND QUALITY ASSURANCE & DATA AND SAFETY MONITORING

The research project is a two-arm randomized clinical trial that includes a single site. This is an uncomplicated, blinded trial, and the risk is considered to be minimal. The University of Illinois at Chicago (UIC) Institutional Review Board (IRB) will review and approve the research before the research is conducted and an IRB-approved data and safety monitoring plan will be documented. Due to the vulnerable study population and the immediate public health impact, a Data Safety and Monitoring Board (DSMB) will be assembled to ensure the safety of the subjects and the validity and integrity of the data generated.

Therefore, the PI and the PI's research team have primary responsibility for monitoring subject safety. The PI is responsible for minimizing research-associated risk. This is done through continuous monitoring of the procedures and through weekly research team meetings. Three senior individuals with expertise in hypertension and clinical trials, external to the research team will serve on the DSMB. Dr. Prendergast, in consultation with the DSMB, will report any adverse events to the Funding Institute and Center. We will attempt to determine the cause of the adverse event and make a determination as to whether the adverse event was related to any study-related activity. When appropriate, we will make changes to the informed consent document and/or study protocol resulting from an adverse event. The following is a list of Adverse Event descriptors that will serve as a guideline to notify IRB and DSMB review boards in the event any TOUCHED study participants experience abnormal symptoms or conditions.

Table 2: Adverse events descriptors

**Study Related Adverse Events**	
**Symptoms or Condition**	**Expanded Comments on Symptom or Condition**
Rash/itching	Covers rash, itching, hives, flushing or similar change in skin. Do not report if symptoms are related to contact with an allergen such as poison ivy or oak.
Angioedema	Covers swelling of the lips, face or tongue.
Cough	Use only for a new cough that is not related to a cold, other infection or seasonal allergy and that follows initiation of a new medication.
Lower extremity edema	Swollen legs or lower extremity edema that is new in onset or substantially worse than usual for the subject.
Lightheadedness or passing out	Lightheadedness, dizziness, passing out, or loss of consciousness
Orthostatic hypotension	Orthostatic hypotension (that is not chronic)

Hypertensive urgency	As indicated by the average blood pressure found at a study visit or a single BP found during a clinic visit that occurred since the last study visit
**Non-study Related Adverse Events**	
**Symptoms or Condition**	**Expanded Comments on Symptom or Condition**
Fever	Do not report if fever is related to a cold or viral infection.
Rhythm disorder	Covers any new rhythm disorder such as tachycardia or a racing heartbeat that is not typical for the subject.
Chest pain	Covers any new development or worsening of chest pain that does not reflect stable angina.
Headache	Do not report if subject has a history of frequent headaches.
Other pain	Other pain that is not related to an injury or chronic condition.
Shortness of breath	Covers shortness of breath, wheezes, stridor, and gasping for breath that is not typical for the subject.
Weight gain	Use for new and unintended weight gain with sudden onset, e.g., related to CHF.
Kidney problem	Covers a new or worsening kidney problem such as acute renal failure or a 20% increase in creatinine level; do not report a kidney infection.
Liver problem	Covers a new or worsening liver problem such as an increase in one or more liver function tests to > 2 times normal.
Nausea or vomiting	Do not report instances related to influenza, other infection or food poisoning.
Other GI problem	Use only for a new GI problem such as diarrhea, constipation, cramping or abdominal pain that is not related to influenza, other infection or food poisoning; do not use to report nausea or vomiting
Neurological change	Use only for a new or worsening neurological change, such as tingling in hands or feet
Trouble walking or falls	Trouble walking or falls
Urinary problem	Urinary problem such as urgency or frequency that is not related to infection
Blood disorder	Bleeding that is not related to or a blood dyscrasia such as leucopenia or thrombocytopenia
Change in lab values	Change in lab values related to a drug side effect such as a marked drop in potassium or sodium or a marked increase in serum creatinine.
Weakness	Covers new onset or worsening of weakness, fatigue, lethargy or other marked decrease in strength
Other	CHECK WITH HEATHER PRENDERGAST BEFORE USING THE ‘Other’ DESCRIPTOR

In the event the “Other” descriptor is to be used to report a specific symptom that is not on the list above the Principal Investigator, Heather Prendergast, must be consulted.

The PI is also responsible for protecting the confidentiality of subjects' data. All documents and information about this study will be kept confidential in accordance with federal, state, and local laws and regulations. Medical records and data generated by the study may be reviewed by the UIC Institutional Review Board, the Office for Human Research Protections, and the National Institutes of Health to assure proper conduct of the study and compliance with federal regulations. The results of this study will be published. If results are published, no subject will be identified by name.

The following procedures are in place:

- We do not collect any subject identifiers that we do not need.
- We remove/destroy subject identifiers as soon as they are no longer needed.
- We restrict physical access to any area or computer system that contains subject identifiers.
- Restrict *electronic* access to any computer system that contains subject identifiers.
- Subject identifiers are stored electronically via REDCap and will **not** be exported with research data that is analyzed. All information collected in this study is confidential and all data will be transformed into anonymously coded identifiers for each subject prior to analysis.
- Subject identifiers and contact information are never distributed outside of University of Illinois at Chicago.

12.0 STATISTICAL CONSIDERATIONS

Outcomes, Analysis Methods and Sample Size

Statistical Analysis Plan: Outcome analyses will be conducted within the UIC Institute for Minority Health virtual environment. Descriptive statistics will be used to assess completeness of study data, normality of outcome measures, and potential covariates as well as to identify potential covariate imbalances between study arms. Based on our pilot study work and our protocol, which emphasizes repeated telephone contacts with participants' post-baseline, we anticipate relatively low loss to follow up at 3 and 6 (at most 20%). For the primary analysis of each study hypothesis we will initially adopt an "intention to treat", (ITT) modality. We will apply the independent samples t-test and Chi-squared test to compare key measures between

treatment arms to ascertain balancing of important measures that may have an influence on the outcome variables. All statistical analysis will be conducted using SAS 9.4 (SAS Institute Inc., Cary, NC, USA) and R version 3.3.1.A brief description of the analytical plan by specific aim follows:

Aim 1: Evaluate the effectiveness of an ED-based E^2 intervention with PACHT-c intervention (arm 2) on the primary outcome of *mean SBP difference* at 6 -months post-intervention compared to usual care (arm 1).

Aim 2: Evaluate the effectiveness of an ED-based E^2 intervention with PACHT-c on the secondary outcome of *mean SBP and DBP differences* at 3-months and 12-months and *mean DBP differences* at 6 months post-intervention compared to usual care.

Aim 3: Examine if the E^2 intervention reduces racial disparities. Specifically, if the intervention is as effective in racial minorities as non-minorities. This will be an exploratory analysis and will primarily focus on changes in cardiovascular risk score/profile. Also included will be an assessment of intervention mediators: primary care engagement, medication adherence, and HTN knowledge in these same patients at 3 and 6 months post randomization-intervention

Aim 1: We hypothesize that the mean change in SBP at 6 months after randomization will be higher in the intervention arm compared to usual care. Linear regression models will be used to compare change in continuous BP between treatment arms at 6 months; mixed effects linear regression models will be used to combine the two-time points into a single model.

Aim 2: We hypothesize that the mean change in SBP and DBP at 3 and 12 months and mean DBP at 6 months after randomization will be higher in the intervention arm compared to usual care. Linear regression models will be used to compare change in continuous BP between treatment arms at 3 months and DBP at 6 months; mixed effects linear regression models will be used to combine the two-time points into a single model.

Aim 3: Change in the cardiovascular risk score at 3 and 6 months will be analyzed to assess racial disparity by testing the interaction between a binary indicator of treatment group and a binary indicator of minority versus non-minority. The distribution of change scores will be examined to determine whether linear regression is appropriate; if not, ordinal categories will be defined and proportional odds logistic regression will be used. Models will be fit at each of 3

and 6 months, and mixed-effects models will be used to combine the two-time points into a single model.

Handling Missing Data: For the missing data analysis an ITT will be implemented. Furthermore, missing data will be handled by using a mixed-effects model approach, which assumes that the data is missing at random and hence will not bias the analysis. If we determine that data may not be randomly missing and thus non-ignorable then two well accepted approaches to handle this situation will be implemented, namely selection models and pattern mixture models.

Other Statistical Considerations: Sampling balance for the key predictors will be checked and adjusted for in the analysis, and their mediating and moderating effects will be examined if needed. We address power for Aims 1 and 2 for unadjusted tests. Adjustment for covariates will decrease power slightly by using up degrees of freedom, but the adjustment will also create more accurate estimates, so the expected power will be similar.

**Effect Size**			**80% Power**		**85% Power**	
**Standardized**	**mmHg***		**Sample Size**		**Sample Size**	
	**DBP**	**SBP**	**N**	**N****	**N**	**N****
**0.20**	**2.40**	**4.20**	**786**	**1049**	**898**	**1198**
**0.25**	**3.00**	**5.25**	**504**	**673**	**576**	**769**
**0.30**	**3.60**	**6.30**	**350**	**467**	**400**	**534**
**0.35**	**4.20**	**7.35**	**258**	**345**	**294**	**393**
**0.40**	**4.80**	**8.40**	**198**	**265**	**178**	**238**
**0.45**	**5.40**	**9.45**	**156**	**209**	**178**	**238**
**0.50**	**6.00**	**10.5**	**126**	**169**	**144**	**193**

***Effect size is defined as difference in means divided by standard deviation. From our pilot study: DBP Standard deviation 12 mmHg; SBP Standard Deviation 21 mmHg. **25% attrition is assumed and thus required sample size, N**, is obtained by dividing calculated sample size, N, by 0.75.**

Sample Size: The primary hypothesis of the study is that the difference in systolic blood pressure from baseline to month 6 is different across the two treatment arms. Testing this hypothesis amounts to testing difference in mean systolic blood pressure between the two treatment groups at 6 months, due to randomization (since the mean SBP at baseline is the same across groups). Thus, sample size estimation is determined by comparing the mean SBP at 6 months using a two-sample, two-sided, 5% significance t-test with 80% and 85% statistical power.

Sample size is calculated first to detect a pre-determined effect size (difference in means divided by standard deviation) and then converted to blood pressure units, namely mmHg. Data from out pilot study resulted in estimates of DBP standard deviation of 12 mmHg and SPB standard deviation of 21 mmHg. For example, the required sample size to detect an effect size of 0.25 (0.25*12 = 3 mmHg in DBP, and 0.25*21 = 5.25 mmHg in SBP) is 504 for 80% power and 576 for 85% power, using a two-sided, two-sample 5% significance t-test. An attrition-adjustment to the calculated sample size give 673 and 770 patients for 80% and 85%, respectively.

The statistical analysis: Mean BPs will be compared across groups using a two-sample, two-sided, 5% significance t-test. The analysis will be then extended to gain statistical power by adjusting the comparison by baseline BP levels. An intention-to-treat analysis will be undertaking by using mixed effects models so that all the available information on each patient is used for the analyses. Further analyses using maximum likelihood estimators for data imputation and weighting will be implemented to better account for missing data. Moreover, key variables (age, sex, race/ethinicity) will be compared across treatment groups and if different will be used in a multivariate mixed effects model.

Patient Accrual: Using the UI Health Enterprise Data Warehouse (EDW), a report was developed to estimate how many patients seen in the ED had elevated BP (Stage 1 ≥140/90 mmHg). The sampling was conducted from April 1, 2014 to December 31, 2014 (9 months). During the sampling period, 8,021 patients had BP readings of at least 140/90 mmHg (29.16 patients per day) as the final ED BP measurement. Approximately 69% of these patients were discharged. The proposed study requires enrollment of 13.9% (770/5534) of potentially eligible UI Health patients over a 4-year period. We are confident that we can meet our recruitment goals.

Data Management: Electronic data entry will utilize REDCap (Research Electronic Data Capture). REDCap is a secure, web-based application providing data quality control through validation options, audit trails and user access controls. Study information within the virtual environment will be available for participant tracking activities. Data quality related to the entry process can be controlled by programming REDCap validation options. Tracking reports will be

reviewed with study staff every two weeks. Statistical analyses prior to the 3, 6, and 12-month outcome evaluations will be limited to quality control, enrollment and dropout reporting, and participant demographics. No interim looks are anticipated, but quality control and data entry errors and omissions will be continuously monitored and reported to staff for remediation. Many of the needed REDCap forms for the TOUCHED proposal were already developed for use for the R56 pilot study.

13.0 REGULATORY REQUIREMENTS

13.1 Informed Consent

Written informed consent will be obtained from all subjects who present to the ED with eligibility criteria, namely elevated blood pressure consistent with stage I hypertension (≥140/90). Patients will be shown a consent video to better streamline and standardize explanation of the study. Consent will be obtained from all subjects interested in participating after the entire study and its risks and hazards have been discussed and all of the subject's questions have been answered. Consent will only be obtained by study staff who have completed Human Subject Research Protection training. In order to minimize the possibility of coercion or undue influence on potential subjects, subjects will only be approached for study consent after they have completed their evaluation in the emergency department and have been identified for discharge. Subjects will also be informed that participation is voluntary and will not affect their relationship with the university/hospital if they decline to participate. Also, patients that are being cared for by the PI or key personnel will not be approached and consented by that individual but rather another member of the study team. Subjects will initially be identified by review of the tracking board listing in the department. The tracking board displays the patients' last name, room number, age, and vital signs. If patients meet the BP requirement (BP ≥140/90) and are slated for discharge from the emergency department, then the study personnel will approach the attending physician caring for the patient for preliminary review of exclusion criteria. At no time during enrollment will any information be placed in the subjects' medical record.

All information collected in this study is confidential and all data will be transformed into anonymously-coded identifiers for each subject prior to analysis. We use a double coding system to maintain the confidentiality of information. Only Dr. Prendergast (PI) and study personnel will be able to link information to subjects. This is necessary because patients will be followed for a total of one year after discharge so participants will need to be tracked during this time. All data will be stored electronically on REDCap.

Data Characteristics:

All data forms will be initially developed and tested on paper; Spanish language versions of participant questionnaires will be prepared and back-translated to English prior to obtaining certified translations; after thorough pilot testing, paper forms are to be converted to equivalent electronic data entry forms with use of REDCap. REDCap (Research Electronic Data Capture) is a secure, web-based application designed to support data capture for research studies, providing: 1) an intuitive interface for validated data entry; 2) audit trails for tracking data manipulation and export procedures; 3) automated export procedures for seamless data downloads to common statistical packages; and 4) procedures for importing data from external sources. At UI Health, REDCap is hosted on secure servers located in and supported by the Institute for Health Research and Policy (IHRP). Project staff will be trained in the use of REDCap for entry of study data. Appropriate logic and limits checking (including cross-form validity checks) will be implemented in REDCap to facilitate accurate and consistent data entry. Where practical, study data will be directly entered into the REDCap database by clinical staff. Alternatively, information can be captured on paper and entered into the system from the forms. Routinely throughout the study the REDCap database will be downloaded into a secure virtual computing environment (virtual servers and virtual workstations secured behind software and hardware firewalls) available through IHRP. Study information within the virtual environment will be available for participant tracking activities such as production of result and reminder letters, scheduling of interim telephone contacts, and scheduling and tracking of follow-up visits. Regular quality control and data cleaning/ error reports will be produced which identify missing, out of range, and questionable values. These reports will be reviewed with study staff every two weeks. Errors will remain on reports until they are

resolved. Statistical analyses prior to the 3 and 6-month outcome evaluations will be limited to quality control, enrollment and drop out reporting and participant demographics. No interim looks are anticipated but quality control and data entry errors and omissions will be continuously monitored and reported to staff for remediation.

13.2 Subject Confidentiality

The PI is responsible for protecting the confidentiality of subjects' data. All data will be entered into electronic database (REDcap). There will be no paper charts/data entry. This will minimize the potential for loss of information and breech of privacy. All of this information will be handled in accordance with HIPAA guidelines.

Patient confidentiality will be maintained through secured, password protection computerized data collection. All documents and information about this study will be kept confidential in accordance with federal, state, and local laws and regulations. Medical records and data generated by the study may be reviewed by the **UIC Institutional Review Board,** and **The Office For Human Research Protections,** to assure proper conduct of the study and compliance with federal regulations. The results of this study will be published. If results are published, no subject will be identified by name.

13.3 **Unanticipated Problems**

Any unanticipated problems will be addressed immediately by the PI and brought to the DSMB. Any unanticipated problems will also be reported and documented in written document to the IRB and sponsor (if applicable).

14.0 REFERENCES

1. Bonds, D.E., et al., *A multifaceted intervention to improve blood pressure control: The Guideline Adherence for Heart Health (GLAD) study.* American heart journal, 2009. **157**(2): p. 278-84.

2. *Racial/Ethnic disparities in the awareness, treatment, and control of hypertension - United States, 2003-2010.* MMWR. Morbidity and mortality weekly report, 2013. **62**(18): p. 351-5.

3. Begley, C.E., et al., *Emergency room use and access to primary care: evidence from Houston, Texas.* Journal of health care for the poor and underserved, 2006. **17**(3): p. 610-24.

4. Levy, P.D. and D. Cline, *Asymptomatic hypertension in the emergency department: a matter of critical public health importance.* Academic emergency medicine : official journal of the Society for Academic Emergency Medicine, 2009. **16**(11): p. 1251-7.

5. Preston, R.A., et al., *Clinical presentation and management of patients with uncontrolled, severe hypertension: results from a public teaching hospital.* Journal of human hypertension, 1999. **13**(4): p. 249-55.

6. Prendergast, H., Colla ,J., Patel, N., Del Rios, M., Marcucci, J.,Scholz R., Ngwang, P., Cappitelli, K.,Daviglus, M., Dudley, S., *Correlation between Subclinical Heart Disease and Cardiovascular Risk Profiles in an Urban Emergency Department Population with Asymptomatic Hypertension: A Pilot Study*, in *submitted for publication*.

7. Kupferman, J.C., et al., *BP Control and Left Ventricular Hypertrophy Regression in Children with CKD.* Journal of the American Society of Nephrology : JASN, 2014. **25**(1): p. 167-74.

8. Aljaroudi, W.A., et al., *Prognostic Value of Diastolic Dysfunction: State of the Art Review.* Cardiology in review, 2013.

9. Berenson, J. and A. Shih, *Higher readmissions at safety-net hospitals and potential policy solutions.* Issue brief, 2012. **34**: p. 1-16.

10. Ndumele, C.D., et al., *Cardiovascular disease and risk in primary care settings in the United States.* Am J Cardiol, 2012. **109**(4): p. 521-6.

11. Baumann, B.M., D.M. Cline, and E. Pimenta, *Treatment of hypertension in the emergency department.* Journal of the American Society of Hypertension : JASH, 2011. **5**(5): p. 366-77.

12. Baumann, B.M., et al., *Evaluation, management, and referral of elderly emergency department patients with elevated blood pressure.* Blood pressure monitoring, 2009. **14**(6): p. 251-6.

13. Baumann, B.M., et al., *Provider self-report and practice: reassessment and referral of emergency department patients with elevated blood pressure.* American journal of hypertension, 2009. **22**(6): p. 604-10.

14. Tanabe, P., et al., *Increased blood pressure in the emergency department: pain, anxiety, or undiagnosed hypertension?* Annals of emergency medicine, 2008. **51**(3): p. 221-9.

15. Statistics, N.C.f.H., *Health, United States, 2013: With Special Feature on Prescription Drugs.* 2014, Hyattsville, MD.

16. Chobanian, A.V., et al., *Seventh report of the Joint National Committee on Prevention, Detection, Evaluation, and Treatment of High Blood Pressure.* Hypertension, 2003. **42**(6): p. 1206-52.

17. Gu, Q., et al., *High blood pressure and cardiovascular disease mortality risk among U.S. adults: the third National Health and Nutrition Examination Survey mortality follow-up study.* Annals of epidemiology, 2008. **18**(4): p. 302-9.

18. Gu, Q., et al., *Association of hypertension treatment and control with all-cause and cardiovascular disease mortality among US adults with hypertension.* American journal of hypertension, 2010. **23**(1): p. 38-45.
19. Burt, V.L., et al., *Trends in the prevalence, awareness, treatment, and control of hypertension in the adult US population. Data from the health examination surveys, 1960 to 1991*. Hypertension, 1995. **26**(1): p. 60-9.
20. Chobanian, A.V., et al., *The Seventh Report of the Joint National Committee on Prevention, Detection, Evaluation, and Treatment of High Blood Pressure: the JNC 7 report.* JAMA : the journal of the American Medical Association, 2003. **289**(19): p. 2560-72.
21. Kramer, H., et al., *Racial/ethnic differences in hypertension and hypertension treatment and control in the multi-ethnic study of atherosclerosis (MESA).* Am J Hypertens, 2004. **17**(10): p. 963-70.
22. Ferdinand, K.C. and A.M. Armani, *The management of hypertension in African Americans.* Critical pathways in cardiology, 2007. **6**(2): p. 67-71.
23. Guzman, N.J., *Epidemiology and management of hypertension in the Hispanic population: a review of the available literature.* American journal of cardiovascular drugs : drugs, devices, and other interventions, 2012. **12**(3): p. 165-78.
24. James, P. A., Oparil, S., Carter, B. L., Cushman, W. C., Dennison-Himmelfarb, C., Handler, J., . . . Ortiz, E. (2014). *2014 Evidence-Based Guideline for the Management of High Blood Pressure in Adults*. Jama, 311(5), 507. doi:10.1001/jama.2013.284427
