# Supplementary material for: Emergency Department–Based Education and mHealth Empowerment Intervention for Hypertension: The TOUCHED Randomized Clinical Trial
Source: JAMA Cardiol. 2025 Apr 23;10(7):657–65. doi: 10.1001/jamacardio.2025.0675 (PMC12019670; doi:10.1001/jamacardio.2025.0675)
Supplement: Supplement 2. — eMethods. eTable 1. Results for the Interactions Between Time and Treatment Arm Predicting Systolic and Diastolic Blood Pressure Using Observed Data eTable 2. Controlled Blood Pressure (Defined as SBP ≤130 and DBP ≤80) by Treatment Arm eTable 3. Multiple Imputation Results eTable 4. Generalized Linear Models for Binary Outcomes Across 2 Time Points [file jamacardiol-e250675-s002.pdf]

Supplementary Online Content

Prendergast H, Kitsiou S, Petzel Gimbar R, et al. Emergency department–based education and mhealth empowerment intervention for hypertension: the TOUCHED randomized clinical trial. *JAMA Cardiol*. Published online April 23, 2025. doi:10.1001/jamacardio.2025.0675

eMethods.

eTable 1. Results for the Interactions Between Time and Treatment Arm Predicting Systolic and Diastolic Blood Pressure Using Observed Data

eTable 2. Controlled Blood Pressure (Defined as SBP ≤130 and DBP ≤80) by Treatment Arm

eTable 3. Multiple Imputation Results

eTable 4. Generalized Linear Models for Binary Outcomes Across 2 Time Points

This supplementary material has been provided by the authors to give readers additional information about their work.

© 2025 Prendergast H et al. *JAMA Cardiol*.

eMethods.

Details about the main analysis

A linear mixed effects multiple regression model with an unstructured covariance pattern is used to model each of systolic and diastolic blood pressure at baseline, 3 months, and 6 months follow-up. Time is modeled as a fixed factor with three categories (baseline, 3 months and 6 months). Randomly assigned treatment arm at baseline is also a fixed factor. The effect of treatment on change in blood pressure is evaluated at 3 months and at 6 months by testing the significance of the interaction between treatment and time equal to 3 and 6 months respectively.

The model for each of SBP and DBP can be written as

$$Y_{ij} = \beta_0 + \beta_1 I_i\{Treatment\ arm\} + \beta_2 I_j\{3\ months\} + \beta_3 I_j\{6\ months\}$$

$$+\beta_4 I_i\{Treatment\ arm\} I_j\{3\ months\} + \beta_5 I_i\{Treatment\ arm\} I_j\{6\ months\} + \varepsilon_{ij}$$

where Y_{ij} is the jth observation of SBP or DBP for subject *i*; $I_i\{Treatment\ arm\}$ equal to 1 if the ith subject was randomized to treatment arm and zero for control arm; $I_j\{3\ months\}$ is equal to 1 if the jth measurement is at 3 months and 0 otherwise; and $I_j\{6\ months\}$ is equal to 1 if the jth measurement is at 6 months at 0 otherwise. The covariance is unstructured with six estimated parameters allowing for correlation within subjects and assuming independence between subjects. A test comparing this model to a model with random intercepts showed significantly better fit for the unstructured model. Models including both random intercepts and slopes were not able to converge. The test of $H_0: \beta_4 = 0$ is used to evaluate the treatment effect at 3 months after baseline and $H_0: \beta_5 = 0$ for the treatment effect at 6 months after baseline.

The SAS code used to fit this model is the following:

```
proc mixed data=all_long method=ml covtest;
  class id a00q022_trial_arm time(ref=first);
  model sbp=a00q022_trial_arm time a00q022_trial_arm*time / s;
  repeated time / type=un subject=id;
  lsmeans a00q022_trial_arm*time;

run;

proc mixed data=all_long method=ml covtest;
  class id a00q022_trial_arm time(ref=first);
  model dbp=a00q022_trial_arm time a00q022_trial_arm*time / s;
  repeated time / type=un subject=id;
```

© 2025 Prendergast H et al. *JAMA Cardiol*.

```
    lsmeans a00q022_trial_arm*time;

  run;
```

where *id* is subject identifier; *a00q022_trial_arm* is randomized treatment arm; *time* is a factor with three categories (baseline, 3 months, 6 months); *sbp* and *dbp* are systolic and diastolic blood pressure. The essential portion of the output, tests results for the interactions between time and treatment arm predicting systolic and diastolic blood pressure, is summarized in supplementary Table 1, as reported in the manuscript.

Sensitivity Analysis 1: Additional Definition of Controlled Blood Pressure

Controlled blood pressure at followup is defined as SBP<=140 and DBP<=90. Addition of a second more stringent definition, SBP<=130 and DBP<=80, is shown below in supplementary Table 2.

To clarify about the analysis presented in Table 2 for binary outcomes, a separate test is done for the treatment effect at each time point based on the 2x2 contingency table for treatment group by outcome. Simple logistic regression with a single binary predictor and the chi-square test of independence are equivalent ways of obtaining the reported p-values.

Baseline Covariates

The original plan was to use an unadjusted model because treatment assignment has been randomly assigned and is not expected to have any systematic relationship with baseline covariates. Also the consensus of the group was to not report statistical tests comparing treatment arms on baseline characteristics.

Instead we use a different strategy of testing for group differences at baseline and including any baseline factors that differ significantly between groups in the multiple regression model as adjustment variables. None of the baseline variables examined were significantly different, so the analysis model remains unadjusted using this strategy (independent samples t-test results - p=.1338 for age, p=.8779 for years of education completed; chi-square test results - p=.9152 for gender, p=.8437 for Black race, p=.1229 for medicare/medicaid yes/no; MH test of trend. p=.7669 for increasing categories of

© 2025 Prendergast H et al. *JAMA Cardiol*.

education completed, p=.8334 across categories of "How well do you get along on your household income?").

Sensitivity Analysis 2: Multiple Imputations

The mixed effects linear regression model assumes missing data is missing at random conditional on all effects in the model. Another approach based on the same assumption is to perform multiple imputations generating several imputed complete data sets, fit the analysis model on each imputed data set, and average the results together.

As a sensitivity analysis, we use PROC MI in SAS to generate imputations based on the same variables used in the analysis model (treatment arm; SBP and DBP at each of baseline, 3 months, and 6 months). Imputations were done using the method of full conditional specification (FCS) for data with arbitrary missing patterns. The SAS code used is:

```
proc mi data=new_all_wide out=out_mi;
  class a00q022_trial_arm;
  var a00q022_trial_arm sbp_bl sbp_3mos sbp_6mos dbp_bl dbp_3mos dbp_6mos;
  fcs outiter=temp_mi discrim;

run;
```

SAS automatically increased the standard number of imputations from 5 to 25 due to the large amount of missing data. Resulting estimates and corresponding standard errors for the main analysis tests, interactions between time and treatment arm, were combined together as described in PROC MIANALYZE documentation reflecting standard methods for accounting for variability due to imputations. Results are summarized in SUPPLEMENTAL TABLE 3.

Analysis 3: Generalized Linear Models for Binary Outcomes

The original plan was to use separate logistic regression models for each time point 3 months and 6 months to evaluate yes/no blood pressure is controlled based on both SBP and DBP, also to evaluate whether or not there was complete follow-up. A separate evaluation at each followup is consistent with our main analysis of blood pressure values.

As an additional analysis we consider instead a single model for each outcome combining results at 3 months and at 6 months using PROC GENMOD in SAS. The method is not the

© 2025 Prendergast H et al. *JAMA Cardiol*.

same as our main analysis with time as a three category factor. In this case there is no baseline outcome (all subjects are not controlled at baseline), time has only two categories, and an interaction with time no longer reflects a treatment effect but instead a difference between treatment effects at the two follow-up points.

Interactions with time are not significant, suggesting perhaps use of a single estimate averaging across the two follow-up points. Results are summarized below in SUPPLEMENTAL TABLE 4.

© 2025 Prendergast H et al. *JAMA Cardiol*.

eTable 1. Results for the interactions between time and treatment arm predicting systolic and diastolic blood pressure using observed data.

Main Results from PROC MIXED					
	Comparisons Between Treatment Arms (Treatment X Time)				
	Estimate	Standard Error	DF	T Value	Pr > |t|
Systolic BP					
Treatment X (3mos-BL)	-4.1921	2.2400	572	-1.87	0.0618
Treatment X (6mos-BL)	-4.8990	2.0943	572	-2.34	0.0197
Diastolic BP					
Treatment X (3mos-BL)	-1.6648	1.3179	572	-1.26	0.2070
Treatment X (6mos-BL)	-2.3323	1.2896	572	-1.81	0.0710

© 2025 Prendergast H et al. *JAMA Cardiol*.

eTable 2. Controlled Blood Pressure (defined as SBP<=130 and DBP<=80) by treatment arm.

	Control Arm		Intervention Arm		Comparison Between Arms				
	N	n (%)	N	n (%)	Odds Ratio	95% CI LL	95% CI UL	Chi-Square	p-value*
BP Controlled, n (%) (SBP≤130 & DBP≤80)									
at 3 months	151	18 (11.9%)	157	19 (12.1%)	1.02	0.51	2.02	0.002	.9610
at 6 months	203	32 (15.8%)	210	35 (16.7%)	1.07	0.63	1.80	0.06	.8034

© 2025 Prendergast H et al. *JAMA Cardiol*.

eTable 3. Multiple Imputation Results

SUPPLEMENTAL TABLE 3. Multiple Imputations Results<br>Results of PROC MIXED Combining 25 Imputed Datasets							
	Comparisons Between Treatment Arms<br>(Treatment X Time)						
	Q-BAR	W-BAR	B	TVAR	DF	t Value	Pr > |t|
Systolic BP							
Treatment X (3mos-BL)	-4.1251	3.1152	2.6845	5.9070	397.4	-1.6973	0.0904
Treatment X (6mos-BL)	-4.6736	3.3761	1.5743	5.0134	442.2	-2.0873	0.0374
Diastolic BP							
Treatment X (3mos-BL)	-1.7127	1.0783	1.1204	2.2434	100.0	1.1435	0.2556
Treatment X (6mos-BL)	-2.2875	1.2654	0.4689	1.7531	112.6	1.7277	0.0868

*Q-BAR = combined point estimate; W-BAR = within-imputation variance; B = between-imputation variance; TVAR = total variance of Q-BAR; test statistic = Q-BAR / sqrt(TVAR).

© 2025 Prendergast H et al. *JAMA Cardiol*.

eTable 4. Generalized Linear Models for Binary Outcomes Across Two Time Points

SUPPLEMENTAL TABLE 4.<br>Generalized Linear Models for Binary Outcomes Across Two Time Points<br>Odds Ratios for Effect of Intervention on Blood Pressure Control							
	Estimates from Model Including Interaction					Estimates from Model Without Interaction	
	3 months		6 months		Interaction with Time	Common Effect for 3 and 6 months	
	OR	p-value	OR	p-value	p-value	OR	p-value
Controlled BP (SBP<=140 and DBP<=90)	1.54	.0562	1.28	.2267	.4559	1.38	.0581
Controlled BP (SBP<=130 and DBP<=80)	0.96	.9000	1.04	.8885	.8325	1.01	.9604
Complete Followup	1.06	.7470	1.07	.7018	.9246	1.06	.6864

© 2025 Prendergast H et al. *JAMA Cardiol*.
