# Supplementary material for: Emergency Department–Based Education and mHealth Empowerment Intervention for Hypertension: The TOUCHED Randomized Clinical Trial
Source: JAMA Cardiol. 2025 Apr 23;10(7):657–65. doi: 10.1001/jamacardio.2025.0675 (PMC12019670; doi:10.1001/jamacardio.2025.0675)
Supplement: Supplement 3. — Data Sharing Statement. [file jamacardiol-e250675-s003.pdf]

Data Sharing Statement

Prendergast. Emergency Department–Based Education and mHealth Empowerment Intervention for Hypertension. *JAMA Cardiol.* Published April 23, 2025.
doi:10.1001/jamacardio.2025.0675

Data
Additional Information: ClinicalTrials.gov Identifier: NCT03749499
Data available: No

Additional Information
Explanation for why data not available: While the TOUCHED data sets are not publicly available at this time, they will be eventually available in a data repository in a de-identified format as proposed to NHLBI.
